# FOXA2-Interacting FOXP2 Prevents Epithelial-Mesenchymal Transition of Breast Cancer Cells by Stimulating E-Cadherin and PHF2 Transcription

**DOI:** 10.3389/fonc.2021.605025

**Published:** 2021-02-25

**Authors:** Yuxiang Liu, Taolin Chen, Mingyue Guo, Yu Li, Qian Zhang, Guixiang Tan, Li Yu, Yongjun Tan

**Affiliations:** State Key Laboratory of Chemo/Biosensing and Chemometrics, College of Biology, Hunan Engineering Research Center for Anticancer Targeted Protein Pharmaceuticals, Hunan University, Changsha, China

**Keywords:** FOXP2 transcription factor, epithelial-mesenchymal transition of breast cancer, FOXA2 transcription factor, E-cadherin, PHF2

## Abstract

FOXP2, a member of forkhead box transcription factor family, was first identified as a language-related gene that played an important role in language learning and facial movement. In addition, FOXP2 was also suggested regulating the progression of cancer cells. In previous studies, we found that FOXA2 inhibited epithelial-mesenchymal transition (EMT) in breast cancer cells. In this study, by identifying FOXA2-interacting proteins from FOXA2-pull-down cell lysates with Mass Spectrometry Analysis, we found that FOXP2 interacted with FOXA2. After confirming the interaction between FOXP2 and FOXA2 through Co-IP and immunofluorescence assays, we showed a correlated expression of FOXP2 and FOXA2 existing in clinical breast cancer samples. The overexpression of FOXP2 attenuated the mesenchymal phenotype whereas the stable knockdown of FOXP2 promoted EMT in breast cancer cells. Even though FOXP2 was believed to act as a transcriptional repressor in most cases, we found that FOXP2 could activate the expression of tumor suppressor PHF2. Meanwhile, we also found that FOXP2 could endogenously bind to the promoter of E-cadherin and activate its transcription. This transcriptional activity of FOXP2 relied on its interaction with FOXA2. Furthermore, the stable knockdown of FOXP2 enhanced the metastatic capacity of breast cancer cells *in vivo*. Together, the results suggested that FOXP2 could inhibit EMT by activating the transcription of certain genes, such as E-cadherin and PHF2, in concert with FOXA2 in breast cancer cells.

## Introduction

Breast cancer is the most common malignant tumor and the leading cause of cancer death among women (1). Metastasis of breast cancer is one of the main reasons for poor survival in patients (2). One of the important mechanisms regulating the invasive behavior of cancer cells is the epithelial-mesenchymal transition (EMT), which represents the conversion of differentiated epithelial cancer cells into migratory mesenchymal cancer cells, leading to cancer invasion, systemic cancer cell dissemination and metastasis (3). Moreover, EMT results in cancer cells avoiding cellular senescence and apoptosis, and participates in the generation and maintenance of cancer stem cells (4), highly consistent with the ability of metastatic cells to initiate new tumors (5). During the progress of EMT, the expression of epithelial markers such as the junction protein E-cadherin is lost and the expression of mesenchymal markers such as Vimentin is up-regulated in cancer cells (6). Gene expression profiling experiments of EMT suggest that many genes adjust in their expression during EMT (7), regulated by a network of signaling pathways from a variety of growth factors [i.e., epidermal growth factor (EGF) (8)] and multiple transcription factors (9, 10).

In our previous studies, transcription factor FOXA2 was confirmed to inhibit EMT in breast cancer cells by regulating the transcription of EMT-related genes and the stable overexpression of FOXA2 abolished breast cancer cell metastasis *in vivo* (11). Thus, we intend to identify FOXA2-interacting proteins from FOXA2-pulled down cell lysates with Mass Spectrometry Analysis in current studies. Interestingly, transcription factor FOXP2, another member of the FOX transcription factor family, has been found to interact with FOXA2. The FOX transcription factor family is widely distributed in various eukaryotes and contains more than 40 mammalian members, which possess a conserved DNA binding domain (DBD) known as Forkhead box/winged helix domain (12). The chromatin immune-precipitation experiment identifies the candidate FOXP2-binding sequence CAAATT as the most probable target for FOXP2 binding in chromatin (13). FOXP2 has been shown to both promote and more often inhibit the transcription of target genes (14). FOXP2 can interact with the co-repressors, such as C-terminal binding protein-1 (CtBP-1) that mediates transcriptional repression primarily through recruitment of histone deacetylases HDAC1/2 (15), to confer its transcriptional repressive properties (16, 17). An increasing amount of evidence supports the repressor role of FOXP2 upon the transcription of its target genes, such as SRPX2/uPAR complex (18) and DLL3 (19), which are involved in oncogenic progression of different types of cancers. On the other hand, FOXP2 has also been reported to activate the transcription of genes, such as the protein-tyrosine kinase SYK that is described as a tumor suppressor in breast cancer cells (20). This transcriptional activation of FOXP2 is often explained by the differential affinity of FOXP2 for DNA binding sites or by the cofactors that interact with FOXP2.

While FOXP2 has first been reported to participate in speech and language development and neuronal development (21, 22), the expression of FOXP2 is observed in multiple adult tissues, such as heart, lung, liver, ovaries, and gut (23, 24). A growing number of evidences have linked FOXP2 to multiple cancers and its dysregulation may play a main role throughout cancer initiation and progression (25), even though it may act as either a tumor-suppressor or a tumor-stimulator depending on the type of cancers studied. For example, its expression is down-regulated in breast cancer (26), hepatocellular carcinoma (27), and gastric cancers (28), in which FOXP2 plays roles as a tumor-suppressor. Conversely, overexpressed FOXP2 has been found in lymphomas (29), neuroblastomas (30), and prostate cancers (31), implicating a pro-oncogenic role of FOXP2 in these cancers. These differences may suggest alternative and tissue-specific roles for FOXP2 as a tumor suppressor or as an oncogene, depending on activated signaling pathways in certain types of cancer. The strong evidence of FOXP2 as a tumor-suppressor role comes from a breast cancer study, in which silencing FOXP2 through miRNA-mediated FOXP2 repression promotes cancer stem cell traits and metastasis in breast cancer cells (32).

In the current study, we identified that FOXP2 interacted with FOXA2, and the expression of FOXP2 was strongly correlated with the epithelial phenotype of breast cancer cells. The stable knockdown of FOXP2 expression promoted the mesenchymal phenotype of breast cancer cells, while the overexpression of FOXP2 inhibited the EMT of breast cancer cells. We confirmed that FOXP2 alone could activate the expression of tumor suppressor PHF2. Meanwhile, FOXP2 could endogenously bind to the promoter of E-cadherin and activate E-cadherin transcription, relying on its interaction with FOXA2. Furthermore, the stable knockdown of FOXP2 enhanced the metastatic capacity of breast cancer cells *in vivo*. Together, the results suggested that FOXP2 could inhibit EMT in breast cancer cells by activating transcription of certain genes, such as E-cadherin and PHF2.

## Materials and Methods

### Cell Culture

The human breast cancer cell lines MCF-7, MDA-MB-231, BT-474, MDA-MB-453, BT-549, ZR-75-30, HCC-1937, MDA-MB-436, MDA-MB-468, and the human kidney cell line HEK-293T were purchased from the Chinese Academy of Sciences Cell Bank. MCF-7, MDA-MB-231, MDA-MB-453, and HEK-293T cells were maintained in DMEM containing 10% fetal bovine serum. MDA-MB-468 and MDA-MB-436 cells were maintained in L-15 medium containing 10% fetal bovine serum. BT-474, BT-549, ZR-75-30, and HCC-1937 cells were maintained in RPMI-1640 containing 10% fetal bovine serum. For the EGF-induced EMT, MCF-7 cells were treated with 100 ng/ml EGF (Invitrogen, USA). For the CTx-induced MET, MDA-MB-468 cells were treated with 50 ng/ml or 100 ng/ml Cholera Toxin (Sigma, USA).

### Mass Spectrometry (MS)

The Avi-FOXA2 DNA fragment was PCR amplified with the following primers: Forward 5′-GGA ATT CAT GTC CGG CCT GAA CGA CAT CTT CGA GGC TCA GAA AAT CGA ATG GCA CGA AAC TAG TAT GCA CTC GGC TTC CAG T-3′ and Backward5′-CCC AAG CTT TTA AGA GGA GTT CAT AAT-3′, using pCMV-FOXA2 (11) as the template. The PCR product was digested and cloned into EcoRI and HindIII sites of pCDNA3.1 plasmid to obtain pAvi-FOXA2. The BirA of *E. coli* was PCR amplified from *E. coli* genomic DNA with the following primers: Forward 5′-CGG ATC CAT GAA GGA TAA CAC CGT GCC ACT G-3′ and Backward5′-CGT CTA GAG GTA GAA GAG GTC AGA CTA CGC-3′. The PCR product was digested and cloned into BamHI and XbaI sites of the lentivirus plasmid vector (33) to obtain pEF1-BirA. MCF-7 cells were plated in 10 cm dishes and transfected with pAvi-FOXA2 (10 μg), pEF1-BirA (5 μg), or both. Forty hours later, the cells were collected, suspended in 100 μl of lysis buffer (150 mM NaCl, 1% NP-40, 50 mM Tris-HCl pH 7.4, protease inhibitor mixture) and incubated for 20 min on ice. The lysates were centrifuged for 15 min at 14,000 g at 4°C and the supernatant containing 200 μg of proteins was incubated with Streptavidin Resin (Sangon Biotech C006390, China) overnight. The resin beads were centrifuged and washed four times with the lysis buffer and subjected to SDS-PAGE, followed by staining with coomassie brilliant blue. Protein samples from SDS-PAGE gel were digested with 10 nmol of MS grade trypsin for 8 h at 37°C and used for mass spectrometry analysis with LTQ Orbitrap Velos Rro (Thermo Fisher Scientific, Swiss).

### Protein Extraction and Western Blotting Assays

To obtain protein extracts, cells were washed with chilled PBS and scraped from culture dishes in lysis buffer (0.5% NP-40, 50 mM Tris-HCl pH 7.5, 100 mM NaCl, 0.1 mM Na_3_VO_4_, 1 mM NaF, 2.5 mM EDTA, 2.5 mM EGTA, 5% Glycerin, 10 mM β-glycerophosphate, protease inhibitor mixture) and stored at -80°C.

To measure certain protein with Western blotting, 50 μg of protein extracts were separated by SDS-PAGE before electro-transferring to PVDF membrane (Millipore, USA). Membranes were probed with specific primary antibodies against proteins of interest and detected with Enhanced Chemiluminescence Plus (Beyotime, China). The following antibodies and dilutions were used: rabbit anti-FOXP2 (1:2,000; Cell Signaling Technology #5337, USA), mouse anti-E-cadherin (1:2,000; Abcam ab1416, UK), rabbit anti-Vimentin (1:1,000; Abcam ab27608, UK), mouse anti-FOXA2 (1:3,000; Abcam ab60721, UK), rabbit anti-PHF2 (1:2,000; Sangon D264123, China), anti-β-actin (1:5,000; Beyotime AA128, China). The signals from the primary antibody were amplified by horseradish peroxidase-conjugated anti-mouse IgG (1:5,000; Sangon D110087, China) or anti-rabbit IgG (1:5,000; Sangon D110058, China).

### Co-Immunoprecipitation (Co-IP)

The His-FOXP2 DNA fragment was PCR amplified with the following primers: Forward 5′-GGA ATT CAT GAT GCA GGA ATC TGC GAC AGA G-3′ and Backward5′-CCC AAG CTT GTG ATG GTG ATG GTG ATG TTC CAG ATC TTC AGA TAA A-3′, using cDNA from MCF-7 cells as the template. The PCR product was digested and cloned into EcoRI and HindIII sites of pcDNA3.1 plasmid to obtain pHis-FOXP2. The FLAG-tagged DNA fragments of FOXA2 or FOXP2 were PCR amplified with the following primers: FLAG-FOXA2 (1-165) Forward 5′-GCT CTA GAA TGG ACT ACA AGG ACG ACG ATG ACA AGA TGC ACT CGG CTT CCA GTA TG-3′ and Backward 5′-CCG GAA TTC TTA CTT TGC GTG CGT GTA GCT G-3′; FLAG-FOXA2 (166-324) Forward 5′-GCT CTA GAA TGG ACT ACA AGG ACG ACG ATG ACA AGC CGC CCT ACT CGT ACA TCT C-3′ and Backward 5′-CCG GAA TTC TTA GCC CCC TCG CTT GTG CTC CTG GCA-3′; FLAG-FOXA2 (325-463) Forward 5′-GCT CTA GAA TGG ACT ACA AGG ACG ACG ATG ACA AGC TGG GAG AGC TGA AGG GGA C-3′ and Backward 5′-CCG GAA TTC TTA AGA GGA GTT CAT AAT GGG CC-3′; FLAG-FOXP2 (1-196) Forward 5′-GCT CTA GAA TGG ACT ACA AGG ACG ACG ATG ACA AGA TGA TGC AGG AAT CTG CGA CAG-3′ and Backward 5′-CCG GAA TTC TCA TTG CTT TCC AGG ATG CTG TTG C-3′; FLAG-FOXP2 (197-408) Forward 5′-GCT CTA GAA TGG ACT ACA AGG ACG ACG ATG ACA AGG CGA AAG AGC AGC AGC AGC A-3′ and Backward 5′-CCG GAA TTC TCA GTG GGT CAT CAT TGC TTG AA-3′; FLAG-FOXP2 (409-633) Forward 5′-GCT CTA GAA TGG ACT ACA AGG ACG ACG ATG ACA AGT TGC ACA TGC GAC CCT CAG AG-3′ and Backward 5′-CCG GAA TTC TCA TGC ATT ATT TAT CAG TCC AGG-3′. The PCR product was digested and cloned into XbaI and EcoRI sites of pcDNA3.1 plasmid.

HEK-293T cells were plated in 10 cm dishes and transfected with pHis-FOXP2 (10 μg) and pAvi-FOXA2 (10 μg) plus pEF1-BirA (5 μg). Two days later, the cells were collected, suspended in 500 μl of lysis buffer (150 mM NaCl, 1% NP-40, 50 mM Tris-HCl pH 7.4, protease inhibitor mixture) and incubated for 20 min on ice. The lysates were centrifuged for 15 min at 14,000 g at 4°C and the supernatant containing 500 μg of proteins was incubated with His-tag Purification Resin (Beyotime P2218, China) or Streptavidin Resin (Sangon Biotech C006390, China) overnight. The beads were centrifuged and washed four times with the lysis buffer and subjected to SDS-PAGE, followed by immunoblotting with anti-FOXP2 (1:2,000; Cell Signaling Technology #5337, USA), anti-FOXA2 (1:1,000; Abcam ab23738, UK), or SA-HRP (1:2000; Beyotime A0312, China).

The co-immunoprecipitation was performed with anti-FLAG affinity gel (Sangon D111139, China) in whole cell extracts of HEK293T cells transfected with FOXP2 expression plasmid (pHis-FOXP2) and FLAG-FOXA2 fragments expression plasmids or FOXA2 expression plasmid (pCMV-FOXA2) and FLAG-FOXP2 fragments expression plasmids. The immunoblotting was performed with anti-FOXP2, anti-FOXA2, or anti-FLAG (Beyotime AF0036, China).

### Immunofluorescence

MCF-7 cells plated on coverslips were washed with PBS, fixed in methanol, and blocked with 3% BSA in 1× PBS plus 0.02% Triton for 1 h at room temperature. Samples were then probed overnight with anti-FOXA2 (1:200; Abcam ab60721, UK) and anti-FOXP2 (1:200; Cell Signaling Technology #5337, USA). After three washes with PBS, cells were incubated at room temperature with FITC-labeled anti-rabbit IgG (1:1,000; Beyotime A0562, China) or Texas Red-labeled anti-mouse IgG (1:1,000; Invitrogen PA1-28626, USA) secondary antibodies for 1 h. Cells were then washed three times with PBS/Triton and mounted on slides with Fluoroshield with DAPI (Beyotime C1002, China). The samples were examined with a TE2000 fluorescence microscope (Nikon Instruments Inc., Japan) (200×).

### Clinical Data Analysis

Clinical breast cancer samples expressing FOXA2 (the BRCA data set, n=394) including both tumor and non-tumor tissue were collected from The Cancer Genome Atlas (TCGA) database (see **Supplementary Table**). The TCGA samples not expressing FOXA2 were excluded for the further analysis in the study. The analysis of FOXP2 levels in the four subgroups (Basal, Her2, LumA, and LumB) of breast cancer and the correlation analysis between FOXA2 and FOXP2 in the BRCA data set were executed by using ggstatsplot package through R project (http://www.r-project.org). For survival curve analysis, two FOXA2-related data sets were first extracted from the BRCA data set, in which the FOXA2^High^ subgroup (n=131) or the FOXA2^Low^ subgroup (n=131) contained either the top 1/3 or the bottom 1/3 of total samples respectively according to the levels of FOXA2 expression. FOXP2-related survival of patients in the FOXA2^High^ and FOXA2^Low^ subgroups was fitted by the “survfit” function, and Kaplan–Meier curves were drawn by the “ggsurv” function in the R package “survival”. The cut-off value of FOXP2 high or low expression was determined by setting “minprop” parameter to 0.3.

### Total RNA Isolation, Blood RNA Isolation, Quantitative Real-Time PCR (qPCR)

Total RNAs from cells or tumors were prepared with Total RNA Kit I (Omega, USA) and blood RNAs from mice were isolated by the Blood RNA Kit (Omega, USA). The cDNAs were synthesized with M-MLV Reverse Transcriptase (Invitrogen, USA) from RNA samples (2 μg) to get 100μl cDNAs. Quantitative real-time PCR (qPCR) was performed by using SYBR Green (Transgen, China) in the realplex2 qPCR system (Eppendorf, Germany) with 1 μl cDNAs as templates in each reaction. The sequences of the primers for sense (S) and antisense (AS) used in non-animal experiments are as follows: hFOXP2-S, 5′-GAA CAC GCA TTG GAT GAC CG-3′ and hFOXP2-AS, 5′-TTG GGA GAT GGT TTG GGC TC-3′; hE-cadherin-S, 5′-CGG GAA TGC AGT TGA GGA TC-3′ and hE-cadherin-AS, 5′-AGG ATG GTG TAA GCG ATG GC-3′; hVimentin-S, 5′-GAG AAC TTT GCC GTT GAA GC-3′ and hVimentin-AS, 5′-GCT TCC TGT AGG TGG CAA TC-3′; 5′-TGT GGG CAT CAA TGG ATT TGG-3′ and hGAPDH-AS, 5′-ACA CCA TGT ATT CCG GGT CAA T-3′; hPHF2-S, 5′-AAA TCT GGG AAG CAG CTG CC-3′ and hPHF2-AS, 5′-ATC TCT TTG GCC AGG TCT TTG-3′; hZEB2-S, 5′-GCG ATG GTC ATG CAG TCA G-3′ and hZEB2-AS, 5′-CAG GTG GCA GGT CAT TTT CTT-3′. The sequence of species-specific primers used in animal experiments is as follows: mCyclophilin-S, 5′-GGC AAA TGC TGG ACC AAA CAC-3′ and mCyclophilin-AS, 5′-TTC CTG GAC CCA AAA CGC TC-3′; hCyclophilin-S, 5′-GCA GAC AAG GTC CCA AAG ACA G-3′ and hCyclophilin-AS, 5′-CAC CCT GAC ACA TAA ACC CTG G-3′; mFoxp2-S, 5′- TTC CAG AGA AGG AAA GAG AG -3′ and mFoxp2-AS, 5′- GAG TCC TAA GTT CAT TCA AC -3′; hFOXP2-S, 5′-ACC AAA GAC ATA CCC TAG TG-3′ and hFOXP2-AS, 5′-ACG TCC CAG ACT GAT GGC AT-3′; mE-cadherin-S, 5′-AGA CAC AAC AGC CCC AAG CC-3′ and mE-cadherin-AS, 5′- GCA ACA GAA TTC AGG AAC AT -3′; hE-cadherin-S, 5′-GTT TCG CTC CAT CGC CCA GG -3′ and hE-cadherin-AS, 5′- TAG CAA GAC CCC ATC TGT AC-3′; mPhf2-S, 5′-TTG CTC CCC CAC CCC CTT CT-3′ and mPhf2-AS, 5′-AGA CTG GAG ACG CGG CTT TC-3′; hPHF2-S, 5′-GTC TTG CCC TGC TGG CCA CAC-3′ and hPHF2-AS, 5′-CAC ACG CTG ACA CGG GAG AA-3′. All qPCR experiments were repeated at least three times.

### Lentivirus Construction and Infection

Three different FOXP2-specific shRNA fragments and a control fragment were cloned into AgeI and EcoRI sites of lentivirus shRNA interference plasmid pMAGic7.1 behind the U6 promoter to obtain pU6-shFOXP2#1, #2, #3, respectively. The sequences for FOXP2-specific shRNA and control fragments primers are as follows: shFOXP2#1-S 5′-CCG GAA CTT GGA AGA ATG CAG TAC TCA AGA GAT ACT GCA TTC TTC CAA GTT TTT TTT G-3′ and shFOXP2#1-AS 5′-AAT TCA AAA AAA ACT TGG AAG AAT GCA GTA TCT CTT GAG TAC TGC ATT CTT CCA AGT T-3′; shFOXP2#2-S 5′-CCG GAG CAA ACA AGT GGA TTG AAC TCA AGA GAT TCA ATC CAC TTG TTT GCT TTT TTT G-3′ and shFOXP2#2-AS 5′-AAT TCA AAA AAA GCA AAC AAG TGG ATT GAA TCT CTT GAG TTC AAT CCA CTT GTT TGCT-3′; shFOXP2#3-S 5′-CCG GTC AAA CAA GTG GAT TGA AAT CTC AAG AGA ATT TCA ATC CAC TTG TTT GTT TTT TG-3′ and shFOXP2#3-AS 5′- AAT TCA AAA AAC AAA CAA GTG GAT TGA AAT TCT CTT GAG ATT TCA ATC CAC TTG TTT GA-3′; shControl-S 5′-CCG GAA CAC CGT TCG AGA CAC GAC TCA AGA GAT CGT GTC TCG AAC GGT GTT TTT TTT G-3′ and shControl-AS 5′-AAT TCA AAA AAA ACA CCG TTC GAG ACA CGA TCT CTT GAG TCG TGT CTC GAA CGG TG TT-3′. The FOXP2-knockdown efficiency of the shRNAs was measured with samples of the varied lentiviral plasmid-transfected MCF-7 cells first. Then, the constructed plasmid (pU6-shFOXP2#1, #2, #3, or pU6-shControl) was cotransfected into 293T cells with two packaging plasmids (pVSVG and Δ8.91) by calcium phosphate transfection to produce lentiviruses. Forty-eight hours post transfection, the medium of 293T was collected and the titration of the virus was measured by flow cytometry (Beckman Coulter, USA). MCF-7 cells were infected with the lentivirus (20 pfu/cell) containing either FOXP2-specific shRNA or shControl to establish cell lines stably expressing shFOXP2 or shControl. Because the lentiviral vector contained an EGFP expression cassette, the infected cells were screened by flow cytometry to obtain cell lines stably expressing EGFP that represented the stable expression of shFOXP2 or shControl. The MCF-7 cell line that stably expresses shFOXP2#2 was used in subsequent experiments.

### Transwell Assays

Cell migration assays were performed by using Transwell migration chambers (8 μm pore size; Corning, USA) according to the vendor’s instructions. Briefly, the cells were trypsinized and 1× 10^4^ cells were plated into the insert of the well. 24 h later, the cells in the insert of each well were removed and the cells under the bottom of the well were stained by 0.1% hexamethylpararosaniline. All experiments were repeated three times. Representative photos were taken using a TE2000 microscope (Nikon Instruments Inc., Japan) (100×) or SMZ1500 stereomicroscope (Nikon Instruments Inc., Japan) (10×). The digital pixel densitometry from at least three different photos was measured with Image-J software (NIH, USA).

### Chromatin Immunoprecipitation (ChIP) Assays

ChIP assays were performed as previously described (11). The following antibodies were used for immunoprecipitation: rabbit anti-FOXP2 (Cell Signaling Technology #5337, USA), rabbit anti-IgG (Millipore PP64, USA). For immunoprecipitation, 2 μg of each antibody was used. The ChIP DNA sample or 1% total input (5 μl) was used in qPCR with the following primers: E-cadherin upstream -760 bp forward: 5′-CGA GAT CGT GCC ACT GCA CTC-3′ and -610 bp backward: 5′-TGG GCT GAA GCG ATC CTC CTG-3′; E-cadherin upstream -414 bp forward: 5′-AGG AGT TCG AGG CTG CAG TG-3′ and -280 bp backward: 5′-TTC TGA TCC CAG GTC TTA GT-3′; PHF2 upstream -1838 bp forward: 5′-CGT GAA GAA CTG AGC CCA GG-3′ and -1693 bp backward: 5′-CGT GAA GAA CTG AGC CCA GG-3′; PHF2 upstream -1519 bp forward: 5′-GAC AAT GGC TTA AGA GTT AC-3′ and -1403 bp backward: 5′-CTG AAT TCC GGT TGT CAC TG-3′.

### Electrophoretic Mobility Shift Assays (EMSA)

FAM-labeled double-strand DNA oligonucleotides were synthesized by Sangon (Shanghai) Co., Ltd, China, based on the sequence 5′-ACT GTT TGT AAA CAG GCT AAT A-3′ from PHF2 upstream region (-1766 bp to -1744 bp) and the sequence 5′-AAA AAT ACA AAC AAA ACA AA-3′ from E-cadherin upstream region (-706 bp to -686 bp), which contained the FOXP2 consensus binding sites. In the binding reactions, 10 μg of nuclear proteins isolated from FOXP2-expressing cells was incubated with 1 pmol of the FAM-labeled probe and 2 μl of 5×binding buffer (Beyotime, China) in a total volume of 10 μl for 30 min at room temperature. The reactions were resolved in 4% native polyacrylamide gel electrophoresis in 0.5×TBE. The dose chosen for the competitive experiments was in the 50× molar excess of the unlabeled oligonucleotides. The oligonucleotides mutated in FOXP2 binding sites (5′-ACT GTT CTG CCC ACC GCT AAT A -3′ for PHF2 or 5′-AAA CAT CCG CAC GCT ACA AA-3′ for E-cadherin) were also used as controls in EMSA experiments. For the supershift analysis, 1 μg of anti-FOXP2 (Cell Signaling Technology #5337, USA) was added to the binding reaction.

### Luciferase Assays

The regions of human E-cadherin or PHF2 promoter were PCR amplified from human genomic DNA with the following primers: hE-cadherin promoter -233 bp to +52 bp: Forward 5′-CCG CTC GAG GGC GCC TTT GTC TTC CCG CT-3′ and Backward 5′-CCC AAG CTT CTT CCG CAA GCT CAC AGG-3′; hE-cadherin promoter -733bp to +60 bp: Forward 5′-CCG CTC GAG TGG GCA AGA CAG AGC GAG AC-3′ and Backward 5′-CCC AAG CTT CTT CCG CAA GCT CAC AGG-3′; PHF2 promoter -2,000 bp to +100 bp: Forward 5′-CGC GCT AGC TGG CTG GAT GTT TAA TTG CT-3′ and Backward 5′-CCG CTC GAG GAC ACA AAA GGG AAT GGA CG-3′. The PCR products were digested and cloned into XhoI and HindIII sites of pGL3 basic Luciferase vector (Promega, USA). The hZEB2 promoter −885 bp to +60 bp plasmid vector was described previously (11). For luciferase assays, MCF-7 cells were transfected with 1.5 μg of different luciferase reporter constructs and a certain amount of pHis-FOXP2, or pCMV-EGFP using Lipofectamine 2000 (Invitrogen, USA), based on different experimental designs. Protein extracts were prepared at 36 h following DNA transfection and the Dual-Luciferase Assay System (Promega, USA) was used to measure Luciferase enzyme activity following the manufacturer’s instruction.

### Transfection of siRNA

Human FOXA2 siRNA (Santa cruz, sc-35569) and control siRNA (Santa cruz, sc-37007) were purchased from Santa Cruz, USA. The siRNA transfection was performed with Lipofectamine 2000 (Invitrogen, USA) according to the manufacturer’s instructions.

### Animal Experiments

All animal experiments were conducted in accordance with institutional animal care and use guidelines, following approval by the Laboratory Animal Center of Hunan, China (Protocol No. SYXK [Xiang] 2008-0001). BalB/c nude mice (female, 4-week old) were purchased from Slac Experimental Animal Company (Changsha, China). To generate mouse models of metastasis of breast cancer cells *in vivo*, MCF-7 cells expressing EGFP or MCF-7 cells stably knockdown FOXP2 were injected into the tail vein of each mouse (2×10^5^ cells/mouse). At day 1, day 30, or day 60 post injection, the mice (n= 6, each group) were sacrificed and blood samples or lung samples were collected. The total RNAs of blood samples or lung samples were isolated and the relative concentration of human tumor cells in the blood or in the lung was determined by qPCR for the mRNA levels of human specific CYCLOPHILIN over the mRNA levels of mouse specific Cyclophilin. The collected lung tissues were fixed overnight in 4% PFA and embedded in paraffin. Sections were stained with hematoxylin and eosin dyes.

### Statistical Analysis

We used Microsoft Excel to calculate SD and determine statistically significant differences between samples and used GraphPad Prism to draw the bar graphs. The asterisks in each graph indicate statistically significant changes with P values calculated by Student T Test: *P < 0.05, **P < 0.01 and ***P < 0.001. P values <0.05 were considered statistically significant.

## Results

### Identification of FOXA2-Interacting Transcription Factor FOXP2

**Figure 1:**
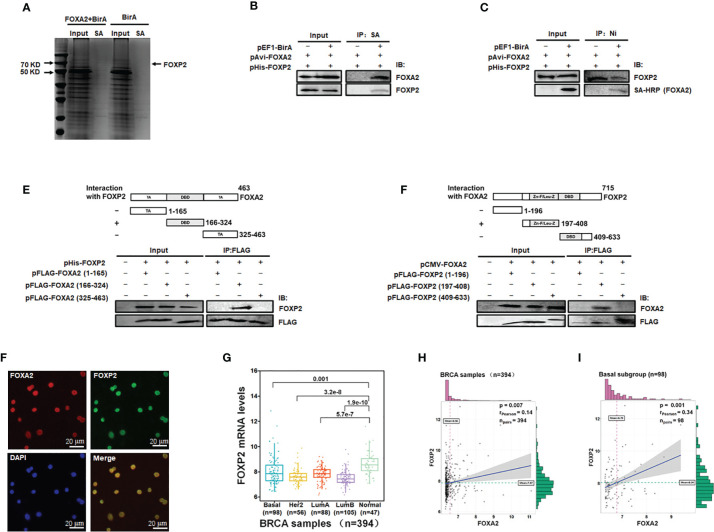
Identification of FOXA2-interacting transcription factor FOXP2 in breast cancer cells. **(A)** The lysates of MCF-7 cells transfected with pAvi-FOXA2, pEF1-BirA, or both, were incubated with streptavidin resin. The pull-down proteins were separated by PAGE and visualized with coomassie brilliant blue staining. The position of FOXP2 was pointed out in the lane of the FOXA2+BirA sample on the gel. **(B, C)** The interaction between FOXA2 and FOXP2 in cells. The lysates of cells transfected with pAvi-FOXA2 and pHis-FOXP2 or pEF1-BirA, were pulled down with streptavidin resin **(B)** or Ni beads **(C)**. Western blotting was performed with anti-FOXA2 antibody, anti-FOXP2 antibody, or streptavidin-conjugated HRP (SA-HRP) respectively. **(D, E)** Mapping the regions mediating FOXA2-FOXP2 interaction in the proteins. The lysates of cells transfected with pHis-FOXP2 plus one of expressing plasmid vectors containing Flag-tagged FOXA2 1-165aa, 166-324aa, and 409-633aa **(D)**, or the lysates of cells transfected with pCMV-FOXA2 plus one of expressing plasmid vectors containing Flag-tagged FOXP2 1-196aa, 197-408aa, 325-463aa **(E)**, were immunoprecipitated with anti-Flag antibody. Western blotting was performed with anti-FOXP2 antibody, anti-FOXA2 antibody, or anti-Flag antibody respectively. The top panel showed a schematic representation of the regions in FOXA2 protein **(D)** and FOXP2 protein **(E)**. **(F)** The interaction of endogenous FOXA2 and FOXP2 was detected by the immunofluorescent microscopy in MCF-7 cells. The staining for FOXA2 (red), for FOXP2 (green), for DAPI (blue), and the merge of the FOXA2 and FOXP2 staining were shown. The cells were pictured by TE2000S (Nikon, 200×). **(G)** The levels of FOXP2 in the four subgroups (Basal, Her2, LumA, and LumB) of breast cancer and normal breast tissue in the clinical breast cancer samples (the BRCA data set, n=394). **(H, I)** The gene expression correlation analysis of FOXA2 and FOXP2 in the BRCA data set (n=394) **(H)** and in the Basal subgroup (n=98) **(I)** were executed by using ggstatsplot package through R project. The pink and green histograms represented the distribution of BRCA samples based on the levels of FOXA2 and FOXP2 expression respectively.

To identify transcription factors interacting with FOXA2 proteins to regulate gene expression, we constructed eukaryotic expression vectors pEF1-BirA and pAvi-FOXA2 and established a biotin tagging system for FOXA2 transcription factor (**Figure S1**). The potential FOXA2-interacting proteins were pulled down with streptavidin resin from MCF-7 cell lysates and separated by PAGE (**Figure 1A**). The samples were analyzed by mass spectrometry and 28 putative FOXA2-interacting proteins were identified (**Table 1**) after filtering out non-specific interactions. Some of the identified proteins, such as HDAC1, had been confirmed to interact with FOXA2 (34), proving the reliability of the analysis. Among the 28 proteins, only three proteins NCOA3 (35), CRTC3 (36), and FOXP2 were found to be transcription factors. FOXP2, as a member of the FOX transcription factor family and a suppressor of tumor metastasis in various human cancers (26, 27), was chosen for subsequent experiments in this study. First, the lysates of MCF-7 cells transfected with pAvi-FOXA2 and pHis-FOXP2 plus pEF1-BirA or not, were pulled down with streptavidin resin (binding to Avi-FOXA2) or Ni beads (binding to His-FOXP2). Following Western blotting, we confirmed that FOXA2 and FOXP2 interacted physically in breast cancer MCF-7 cells (**Figures 1B, C**). Subsequently, we mapped the region mediating FOXA2-FOXP2 interaction in the proteins, using different Flag-tagged fragments of the two proteins for co-imnunoprecipitation assays. FOXA2 protein was divided to three regions including 1-165aa (the N-terminal transcription activation domain), 166-324aa (the DNA binding domain), 325-463aa (the C-terminal transcription activation domain), and FOXP2 protein was divided to three regions including 1-196aa (the N-terminus), 197-408aa (the zinc-finger/leucine zipper motif), 409-633aa (the DNA binding domain plus the C-terminus). The 166-324aa region of FOXA2 and the 197-408aa region of FOXP2 were identified to participate in the interaction between the two proteins (**Figures 1D, E**). The interaction of endogenous FOXA2 and FOXP2 was further confirmed with immunofluorescence assay, which showed that FOXA2 and FOXP2 co-localized in the nucleus of MCF7 cells (**Figure 1F**). Then we analyzed the levels of FOXP2 expression in the clinical breast cancer samples (n=394) from TCGA (**Supplementary Table**) and found that the levels of FOXP2 in all four breast cancer subgroups (Basal, Her2, LumA, and LumB) were lower than that in the normal group (**Figure 1G**). Based on this data set, we observed a weak correlation between the expressions of FOXA2 and FOXP2 in the samples (R=0.14, p=0.007), while a moderate correlation between their expressions was further found in the subgroup of basal breast cancer especially (n=98, R=0.34, p=0.001) (**Figures 1H, I**). Together, these results confirmed that the FOXA2-FOXP2 interaction occurred in breast cancer cells, implicating the involvement of the two proteins in regulating the cancer cells.

**Table 1:** List of candidate FOXA2-interacting proteins from the mass spectrometry screens.

UniProt Accession	Description
O15409-8	**Isoform 8 of Forkhead box protein P2 Gene name=FOXP2**
Q13085	Acetyl-CoA carboxylase 1Gene name=ACACA
P06493	Cyclin-dependent kinase 1 Gene name=CDK1
Q6Y7W6-4	Isoform 3 of PERQ amino acid-rich with GYF domain-containing protein 2 Gene name=GIGYF2
Q5JSZ5	Protein PRRC2B Gene name=PRRC2B
Q9P0J7	E3 ubiquitin-protein ligase KCMF1 Gene name=KCMF1
Q9HCC0-2	Isoform 2 of Methylcrotonoyl-CoA carboxylase beta chain, mitochondrial Gene name=MCCC2
F8W8T8	Acetyl-CoA carboxylase 2 Gene name=ACACB
P43243	Matrin-3 Gene name=MATR3
B1AKL4	Eukaryotic translation initiation factor 4E transporter Gene name=EIF4ENIF1
C9J2P2	DnaJ homolog subfamily B member 6 (Fragment) Gene name=DNAJB6
Q9Y5V3	Melanoma-associated antigen D1 Gene name=MAGED1
Q5T4S7-3	Isoform 3 of E3 ubiquitin-protein ligase UBR4 Gene name=UBR4
Q13547	Histone deacetylase 1 Gene name=HDAC1
Q9Y6Q9-5	Isoform 5 of Nuclear receptor coactivator 3 Gene name=NCOA3
E7EUY0	DNA-dependent protein kinase catalytic subunit Gene name=PRKDC
D6RD67	Methylcrotonoyl-CoA carboxylase beta chain, mitochondrial (Fragment) Gene name=MCCC2
Q6UUV7-3	Isoform 3 of CREB-regulated transcription coactivator 3 Gene name=CRTC3
P61962	DDB1- and CUL4-associated factor 7 Gene name=DCAF7
P52594-2	Isoform 2 of Arf-GAP domain and FG repeat-containing protein 1 Gene name=AGFG1
H3BRH3	Zinc finger CCCH domain-containing protein 18 (Fragment) Gene name=ZC3H18
H0Y720	Trinucleotide repeat-containing gene 6B protein (Fragment) Gene name=TNRC6B
O43347	RNA-binding protein Musashi homolog 1 Gene name=MSI1
Q14677	Clathrin interactor 1 Gene name=CLINT1
O43175	D-3-phosphoglycerate dehydrogenase Gene name=PHGDH
E7ERW8	Protein diaphanous homolog 1 Gene name=DIAPH1
Q86X55-1	Isoform 1 of Histone-arginine methyltransferase CARM1 Gene name=CARM1

### FOXP2 Inhibited the EMT of Breast Cancer Cells

**Figure 2:**
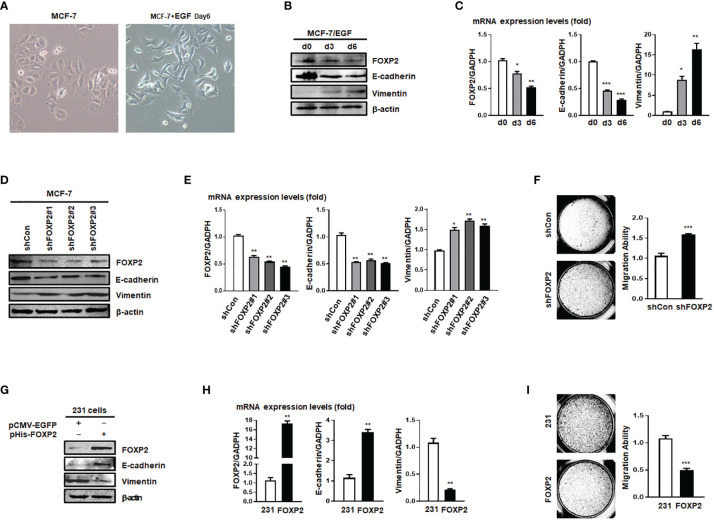
FOXP2 inhibited the EMT of breast cancer cells. **(A)** The changes of the morphology at day 6 post epidermal growth factor (EGF) treatment (100 ng/ml) in MCF-7 cells were pictured by TE2000S (Nikon, 200×). **(B, C)** The expression of FOXP2 was downregulated during EGF-induced epithelial-mesenchymal transition (EMT) in MCF-7 cells. MCF-7 cells were treated with EGF (100 ng/ml) and harvested at different time points (day 0, day 3, day 6) post the treatment. The levels of FOXP2, E-cadherin, and Vimentin were examined by Western blotting **(B)** and qPCR **(C)**, respectively. **(D–F)** Knockdown of FOXP2 enhanced EMT in MCF-7 cells. MCF-7 cells were infected with one of lentiviral vectors expressing various FOXP2-specific shRNA (Lv-shFOXP2#1, #2, #3) or the control lentiviral vector (Lv-shCon). The stable FOXP2-knockdown MCF-7 cells were selected and harvested for protein and total RNA preparation. The levels of FOXP2, E-cadherin, and Vimentin were examined by Western blotting **(D)** and qPCR **(E)**. The migration ability of Lv-shCon-infected MCF-7 cells (shCon) and Lv-shFOXP2#2-infected cells (shFOXP2) was measured by the Transwell invasion test and the statistical data are shown on the right **(F)**. **(G–I)** Overexpression of FOXP2 abolished the mesenchymal phenotype of MDA-MB-231 cells. MDA-MB-231 cells were transfected with pHis-FOXP2 or the control vector pCMV-EGFP. The levels of FOXP2, E-cadherin, and Vimentin were examined by Western blotting **(G)** and qPCR **(H)**. The migration ability of control vector transfected MDA-MB-231 cells (231) and FOXP2-expressing MDA-MB-231 cells (FOXP2) was measured by the Transwell invasion test and the statistical data are shown on the right **(I)**. The asterisks indicate statistically significant changes: *P ≤ 0.05, **P ≤ 0.01, and ***P ≤ 0.001.

The expression levels of FOXP2 varied among different breast cancer cell lines (**Figure S2**), in which the epithelial-type cell lines such as MCF-7 and HCC1397 exhibited higher levels of FOXP2 than that of the mesenchymal-type cell lines such as MDA-MB-231, MDA-MB-453, and MDA-MB-436. This variation of FOXP2 levels matched the different FOXA2 levels among the cell lines mentioned above (**Figure S2**) and implicated that FOXP2 might regulate the epithelial phenotype of the cancer cells. The prediction was first supported by the EGF-induced EMT of MCF-7 cell model in which the epithelial MCF-7 cells acquired a mesenchymal morphology at day 6 post EGF treatment (**Figure 2A**). Both the protein and mRNA levels of FOXP2 were decreased by the EGF treatment, correlated to the decreased levels of the epithelial marker E-cadherin and the increased levels of the mesenchymal marker Vimentin (**Figures 2B, C**). To test whether FOXP2 was required for the epithelial phenotype of breast cancer cells, we used RNA interference strategy to knock down the levels of FOXP2 in MCF-7 cells. The transfection of the three constructed FOXP2-specific shRNA expression plasmids (pU6-shFOXP2#1, #2, #3) resulted in a significant but similar decrease of FOXP2 mRNA and protein levels in MCF-7 cells, among them shFOXP2#2 performing slightly better (**Figure S3**). For further analyzing the roles of FOXP2 in EMT with *in vitro* and further *in vivo* experiments, we infected MCF-7 cells with the lentiviruses expressing various FOXP2-specific shRNA (#1, #2, #3) and selected the FOXP2 stable-knockdown clones of MCF-7 cells through the lentiviral vector-expressed EGFP. FOXP2-knockdown MCF-7 cells exhibited the spindle-like morphology compared with control lentivirus-infected MCF-7 cells (**Figure S4**). Consequently, the knockdown of FOXP2 also decreased the expression of E-cadherin and resulted in the increased expression of Vimentin in the cells (**Figures 2D, E**). In addition, the migration ability of FOXP2-knockdown cells was dramatically strengthened when measured by Transwell invasion test (**Figure 2F**). To test whether FOXP2 abolished the phenotype of mesenchymal breast cancer cells, we transfected the FOXP2-expression plasmids into MDA-MB-231 cells. We found that the overexpression of FOXP2 resulted in the elevation of E-cadherin levels and the reduction of Vimentin levels (**Figures 2G, H**) and decreased the migration ability of MDA-MB-231 cells (**Figure 2I**). Same experiments were also performed with HCC1397 cells (representing epithelial breast cancer cells) and MDA-MB-436 cells (representing mesenchymal breast cancer cells) and the similar phenomena of FOXP2 inhibiting the breast cancer cell EMT were observed (**Figure S5**).

### FOXP2 Stimulated the Expression of PHF2 During MET of Breast Cancer Cells

**Figure 3:**
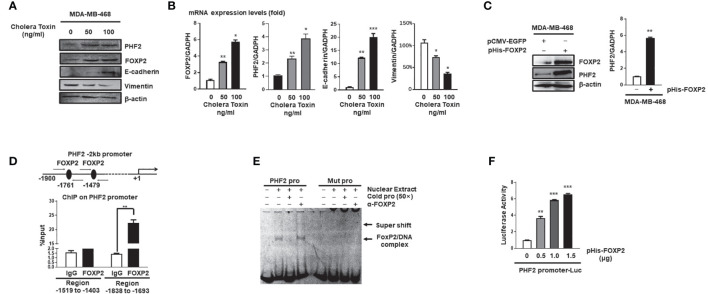
FOXP2 stimulated the expression of PHF2 during MET of breast cancer cells. **(A, B)** The expression levels of PHF2 and FOXP2 were upregulated during CTx-induced MET of MDA-MB-468 breast cancer cells. MDA-MB-468 cells were treated with different concentrations of CTx (50 or 100 ng/ml) for 7 days. The levels of FOXP2, PHF2, E-cadherin, and Vimentin were examined by Western blotting **(A)** and qPCR **(B)**, respectively. **(C)** The overexpression of FOXP2 stimulated PHF2 expression in MDA-MB-468 cells. MDA-MB-468 cells were transfected with pHis-FOXP2 or pCMV-EGFP. The levels of FOXP2 and PHF2 were examined by Western blotting and qPCR. **(D)** FOXP2 bound to the endogenous promoter of PHF2. Gene sequence analysis was performed to predict positions of putative FOXP2 binding sites in -2 kb human PHF2 promoter and the primers for ChIP assays were designed. The chromatin of MCF-7 cells was cross-linked, sonicated, and immunoprecipitated (IP) with either FOXP2 antibody or rabbit IgG. The amount of promoter DNA associated with the IP chromatin was measured by qPCR with primers specific to PHF2 promoter regions -1,838 bp to -1,693 bp and -1,519 bp to -1,403 bp. **(E)** FOXP2 bound to PHF2 promoter region -1,765 bp to -1,743 bp. The nuclear extracts were prepared from pHis-FOXP2-transfected cells and used for EMSAs with a FAM-labeled DNA probe synthesized from PHF2 promoter sequence -1,765 bp to -1,743 bp (PHF2 pro). The unlabeled probe (50×) or 1 μg of FOXP2 antibody (α-FOXP2) was added to the reaction to show the specificity of FOXP2/DNA complex formation. EMSAs with a FAM-labeled mutated probe were also performed (Mut pro). **(F)** The PHF2 promoter was activated by FOXP2 in breast cancer cells. A luciferase reporter plasmid (1.5 μg) containing the fragment of -2,000 bp to +60 bp of PHF2 promoter and loading control pRL-CMV luciferase reporter plasmid (20 ng) was transfected into MDA-MB-468 cells with different amounts of pHis-FOXP2 (0, 0.5, 1.0, and 1.5 μg, balanced with the different amounts of empty expression vector). Protein lysates were prepared at 48 h following transfection and then used to measure dual luciferase enzyme activities. The asterisks indicate statistically significant changes: *P ≤ 0.05, **P ≤ 0.01, and ***P ≤ 0.001.

Mesenchymal-epithelial transition (MET) of cancer cells was a reverse process of EMT and Cholera Toxin (CTx), a protein complex secreted by the bacterium *Vibrio cholerae* (37), was able to induce MET in certain breast cancer cell lines such as MDA-MB-468 but not MDA-MB-231 (38). We established the CTx-induced MET model of MDA-MB-468 cells, in which the MET progression was confirmed by the increase in E-cadherin levels and the decrease in Vimentin levels of the cells (**Figures 3A, B**). As predicted, the FOXP2 expression was induced during this CTx-induced MET progression (**Figures 3A, B**). PHF2 (Plant homeodomain finger 2), a demethylase participating in epigenetic regulation of gene expression through demethylation of histone H3K9m3 (39), was also stimulated in this MET model (**Figures 3A, B**). PHF2 was considered as a tumor suppressor because of its decreased levels in various tumor tissues (40) and could up-regulate certain epithelial genes, leading breast cancer cells to acquire epithelial phenotypes (38). We noticed the correlation of the increased expression of FOXP2 and PHF2, and then found that the overexpression of FOXP2 alone was able to stimulate both the mRNA and protein levels of PHF2 dramatically in MDA-MB-468 cells (**Figure 3C**). To analyze whether FOXP2 stimulated the PHF2 transcription directly, we scanned -2kb promoter region of human PHF2 gene with the FOXP2 DNA binding consensus sequence and found multiple putative FOXP2 binding sites at the regions -1,519 bp to -1,403 bp and -1,838 bp to -1,693 bp in this promoter. ChIP assays confirmed that FOXP2 bound to endogenous PHF2 promoter at regions around -1,838 bp to -1,693 bp but not on another tested region in the cells (**Figure 3D**). EMSAs were performed to confirm the binding of FOXP2 at the PHF2 promoter with a FAM-labeled DNA probe synthesized from the PHF2 promoter region from -1,765 bp to -1,743 bp and nuclear extracts containing FOXP2 proteins. We found that the probe could form a DNA/protein complex in EMSAs with FOXP2, and the addition of either an unlabeled probe (50×) or FOXP2-specific antibody disturbed the formation of the FOXP2/DNA complex or resulted in a supershift complex, whereas the FAM-labeled mutated probe could not form the FOXP2/DNA complex (**Figure 3E**). To further confirm FOXP2 stimulating the PHF2 promoter, we constructed a luciferase reporter plasmid containing the fragment of −2,000 bp to +60 bp of PHF2 promoter. This PHF2 promoter was activated by FOXP2 in a dosage-dependent manner in MCF-7 cells (**Figure 3F**). These results demonstrated that FOXP2 might inhibit the EMT of breast cancer cells by directly stimulating the transcription of epithelial-driven gene PHF2, contrary to the general notion of FOXP2 mainly acting as a transcriptional repressor.

### The Activation of the E-Cadherin Promoter by FOXP2 Relied on the Participation of FOXA2

**Figure 4:**
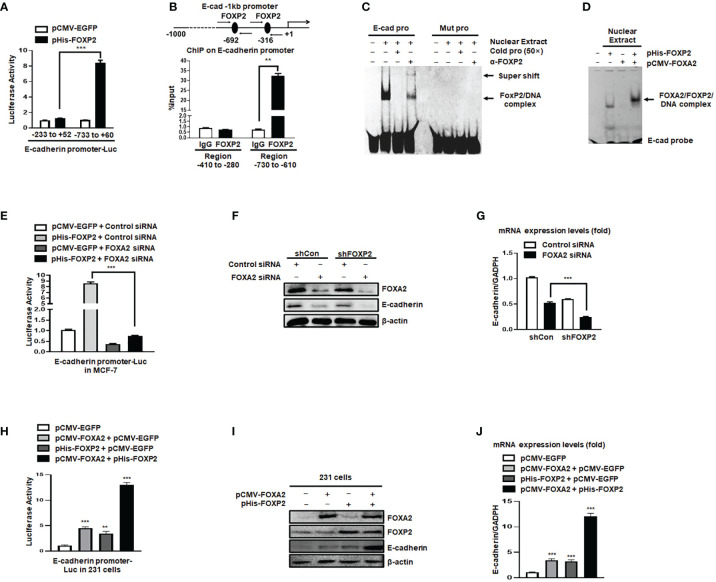
The activation of the E-cadherin promoter by FOXP2 relied on the participation of FOXA2. **(A)** The E-cadherin promoter was activated by FOXP2 in breast cancer cells. A luciferase reporter plasmid (1.5 μg) containing the fragment of -733bp to +60 bp of E-cadherin promoter or a control luciferase reporter plasmid (1.5 μg) containing the fragment of -233 bp to +52 bp of E-cadherin promoter and loading control pRL-CMV luciferase reporter plasmid (20 ng) were transfected into MCF-7 cells with pHis-FOXP2 or pCMV-EGFP (1 μg). **(B)** FOXP2 bound to the endogenous E-cadherin promoter. Gene sequence analysis was performed to predict positions of putative FOXP2 binding sites in -1 kb human E-cadherin promoter and the primers for ChIP assays were designed. The chromatin of MCF-7 cells was cross-linked, sonicated, and immunoprecipitated (IP) with either FOXP2 antibody or rabbit IgG. The amount of promoter DNA associated with the IP chromatin was measured by qPCR with primers specific to E-cadherin promoter regions -760 bp to -610 bp and -414 bp to -280 bp. **(C)** FOXP2 bound to E-cadherin promoter region -701 bp to -691 bp. Nuclear extracts were prepared from pHis-FOXP2-transfected cells and used for EMSAs with a FAM-labeled DNA probe synthesized from E-cadherin promoter sequence -706 bp to −686 bp (E-cad pro). The unlabeled probe (50×) or 1 μg of FOXP2 antibody (α-FOXP2) was added to the reaction to show the specificity of FOXP2/DNA complex formation. EMSAs with a FAM-labeled mutated probe were also performed (Mut pro). **(D)** FOXP2 mediated the binding of FOXA2 to the E-cadherin promoter. Nuclear extracts were prepared from pHis-FOXP2-transfected, pCMV-FOXA2-transfected, or both-transfected cells (48 h) and used for EMSAs with a FAM-labeled DNA probe synthesized from E-cadherin promoter sequence -706 bp to -686 (E-cad probe). **(E)** Knockdown of FOXA2 abolished the activation of the E-cadherin promoter by FOXP2. A luciferase reporter plasmid (1.5 μg) containing the fragment of -733 bp to +60 bp of E-cadherin promoter and loading control pRL-CMV luciferase reporter plasmid (20 ng) were transfected into MCF-7 cells with the pHis-FOXP2 or pCMV-EGFP (1 μg) plus FOXA2 siRNA or Control siRNA (200 nM). Protein lysates were prepared at 36 h following transfection and used to measure dual luciferase enzyme activities. **(F, G)** The knockdown of FOXP2 and FOXA2 together showed a synergistic repression of the E-cadherin expression in breast cancer cells. The shCon MCF-7 cells and shFOXP2 MCF-7 cells were transfected with Control siRNA or FOXA2 siRNA (200 nM) and examined by qPCR for mRNA levels **(F)** and by Western blotting for protein levels **(G)**. **(H)** Overexpression of FOXA2 enhanced the activation of the E-cadherin promoter by FOXP2 in MDA-MB-231 cells. A luciferase reporter plasmid (1.5 μg) containing the fragment of -733 bp to +60 bp of E-cadherin promoter and loading control pRL-CMV luciferase reporter plasmid (20 ng) were transfected into MDA-MB-231 cells with the pCMV-FOXA2 (1 μg), pHis-FOXP2 (1 μg), or both (balanced with the pCMV-EGFP vector). Protein lysates were prepared at 36 h following transfection and used to measure dual luciferase enzyme activities. **(I, J)** The overexpression of FOXP2 and FOXA2 together showed a synergistic activation of the E-cadherin expression in MDA-MB-231 cells. The MDA-MB-231 cells were transfected with FOXA2 expression vector (pCMV-FOXA2), FOXP2 expression vector (pHis-FOXP2), or both (balanced with the pCMV-EGFP vector) and examined by Western blotting for protein levels **(I)** and by qPCR for mRNA levels **(J)**. The asterisks indicate statistically significant changes: **P ≤ 0.01, ***P ≤0.001.

The expression of the junction protein E-cadherin, as a typical epithelial cell marker, decreased during EMT and the decline of E-cadherin’s functions promoted the EMT progression (41). In this study, we found that the levels of E-cadherin relied on FOXP2 in breast cancer cells (see **Figure 2**). We further investigated whether FOXP2 could stimulate the transcription of E-cadherin. In the dual-luciferase reporter assays, FOXP2 was able to activate the -733 bp to +60 bp region of E-cadherin promoter in MCF-7 cells (**Figure 4A**), while the knockdown of FOXP2 resulted in the down-regulated transcriptional activities of the promoter (**Figure S6**). When this promoter was scanned with the consensus FOXP2-DNA binding sequence, we identified multiple putative FOXP2 binding sites at the regions -692 bp to -681 bp and -316 bp to -305 bp. ChIP assays confirmed that FOXP2 bound to endogenous E-cadherin promoter at regions around -760 bp to -610 bp but not another tested region (**Figure 4B**). Consistent with this finding, EMSAs showed that a FAM-labeled DNA probe, synthesized from the E-cadherin promoter region from -706 bp to -686 bp, could form a DNA/protein complex with FOXP2, and the addition of either an unlabeled probe (50×) or FOXP2-specific antibody disturbed the formation of the FOXP2/DNA complex or resulted in a supershift complex, whereas the FAM-labeled mutated probe could not form the FOXP2/DNA complex (**Figure 4C**). These results suggested that the E-cadherin promoter was activated by FOXP2. To test whether the interaction between FOXP2 and FOXA2 affected the transcription of E-cadherin, we performed an additional EMSA experiment, in which the nuclear extracts containing overexpressed FOXP2 only, overexpressed FOXA2 only, or overexpressed FOXP2 and FOXA2 together, were used. We found that FOXP2 and FOXA2 together formed complexes binding to the DNA probe but FOXA2 alone could not bind to the probe (**Figure 4D**), implicating that the mechanism of FOXP2 stimulating the E-cadherin transcription might involve its interaction with FOXA2. This speculation was further supported by the evidence that the knockdown of FOXA2 could significantly decrease the activation of FOXP2 on E-cadherin promoter (**Figure 4E**). Furthermore, the simultaneous knockdown of FOXP2 and FOXA2 synergistically inhibited the expression of E-cadherin in MCF-7 cells (**Figures 4F, G**). When tested in MDA-MB-231 cells possessing low endogenous levels of FOXA2, FOXP2 showed a weaker stimulation on E-cadherin promoter than that in MCF-7 cells (**Figure S7**). The overexpression of FOXP2 and FOXA2 together synergistically activated the expression of E-cadherin in MDA-MB-231 cells (**Figures 4H–J**), resulting in a noticeable morphological change of the cells at the same time (**Figure S8**). Together, these observations suggested that FOXP2 could bind to the E-cadherin promoter and stimulate the transcription of E-cadherin through the FOXP2-FOXA2 interaction in breast cancer cells.

### Stable Knockdown of FOXP2 Activated Migration Capability of Breast Cancer Cells *In Vivo*

**Figure 5:**
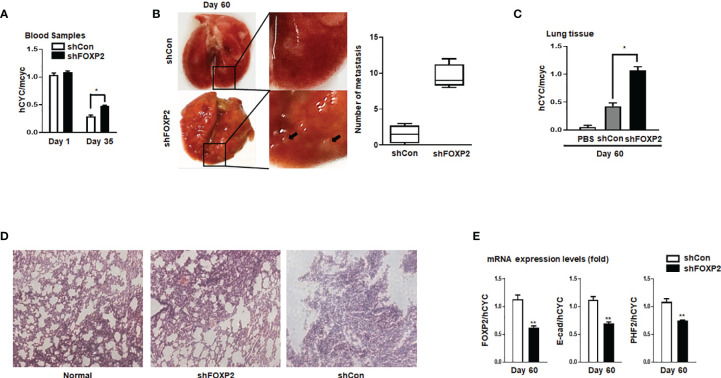
Stable knockdown of FOXP2 stimulated the metastasis of breast cancer cells *in vivo*. The metastasis models of human breast cancer cells in nude mice were created *via* tail vein injection of control lentivirus-infected MCF-7 (shCon) cells or FOXP2-knockdown MCF-7 cells (shFOXP2) (2×10^5^ cells/mouse). The mice (n=12 for each group) were sacrificed at day 1, day 35, and day 60 post-injection. **(A)** The stable knockdown of FOXP2 in cells increased the number of circulating tumor cells in the blood of model mice. Total RNAs of blood of each mouse were isolated at day 1 and day 35 post-injection and the relative concentration of human tumor cells in blood was determined by qPCR for the mRNA levels of human specific CYCLOPHILIN over the mRNA levels of mouse specific Cyclophilin. **(B, C)** Increased tumor formation was found in the lungs of mice injected with shFOXP2 cells. Representative photographs of the lung of the two groups of mice day 60 post-injection were shown, and the statistical data of the tumor foci number are presented on the right **(B)**. Total RNAs of the lung of each mouse were isolated at day 60 post-injection and the relative concentration of human tumor cells in lung was determined by qPCR for the mRNA levels of human specific CYCLOPHILIN over the mRNA levels of mouse specific Cyclophilin **(C)**. **(D)** H&E staining histological images of the lung of the two groups of mice. **(E)** The expression of exogenous human FOXP2, E-cadherin, and PHF2 in lungs of nude mouse metastasis models of human breast cancer cells. The total RNAs of lungs of different groups were isolated and the ratio of mRNA levels of human FOXP2, E-cadherin and PHF2 over human CYCLOPHILIN were determined by qPCR. The asterisks indicate statistically significant changes: *P ≤0.05, **P ≤ 0.01.

**Figure 6:**
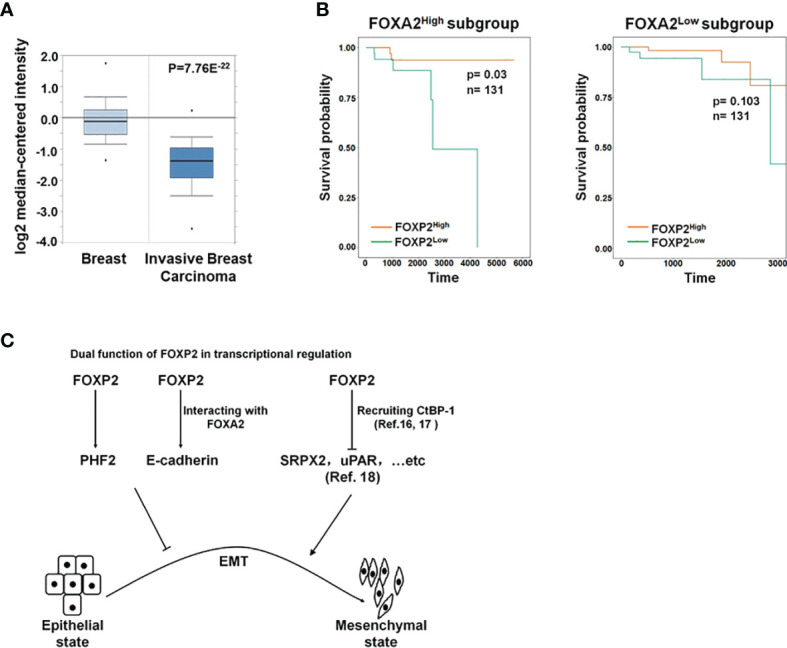
The roles of FOXP2 in EMT of breast cancer cells. **(A)** The Oncomine boxplots of FOXP2 levels were analyzed between invasive breast tissues and normal breast tissues from the oncomine.org website. The p-value of the TCGA RNA-seq data of normal breast samples (n=61) versus invasive breast carcinoma samples (n=76) was 7.76E^-22^. **(B)** The FOXP2-improved survival in breast cancer patients relied on the participation of FOXA2. The FOXA2^High^ subgroup (n=131) or the FOXA2^Low^ subgroup (n=131) contained either the top 1/3 or the bottom 1/3 of the collected BRCA data set (n=394), respectively. FOXP2-related survival of patients in the FOXA2^High^ and FOXA2^Low^ subgroups was fitted by the “survfit” function, and Kaplan–Meier curves were drawn by the “ggsurv” function in the R package “survival”. **(C)** The roles of FOXP2 in epithelial-mesenchymal transition (EMT) of breast cancer cells. FOXP2 prevented EMT of breast cancer cells by regulating the transcription of multiple EMT-related genes: FOXP2 could bind to certain promoters and stimulate the transcription of genes such as PHF2 and E-cadherin. The transcriptional activation by FOXP2 could be mediated by FOXA2. FOXP2 could also repress the transcription of certain genes such as SRPX2 and uPAR, through recruiting co-repressors such as CtBP-1.

To further investigate the functions of FOXP2 on the EMT of breast cancer cells *in vivo*, we generated a metastasis model in nude mice *via* the tail vein injection of the FOXP2-specific shRNA#2 lentivirus-infected MCF-7 cells (shFOXP2) or control lentivirus-infected MCF-7 cells (shCon) (n=12 for each group). The mice were sacrificed at days 1, day 30, and day 60 post-injection, in which the total RNAs of the blood of each mouse were isolated from day 1 and day 30 groups (n=4) and the total RNAs of the lung tissue of each mouse were isolated from day 60 groups (n=4). The relative concentration of circulating human tumor cells in the blood or the lung tissue were determined by the mRNA levels of human-specific CYCLOPHILIN (Gene Symbol: PPIA) compared with those of mouse-specific Cyclophilin (11, 42). The stable knockdown of FOXP2 in MCF7 cells increased the number of circulating tumor cells in the blood of tested mice of day 30 groups (**Figure 5A**). An elevated metastasis and tumor formation were found in the lungs of shFOXP2 cell-injected mice at day 60 (**Figure 5B**). The knockdown of FOXP2 enhancing the lung metastasis of the cells was further observed by EGFP fluorescence intensity in the isolated tissues (**Figure S9A, B**). The amount of metastasized tumor cells in the lungs of day 60 groups was also measured by the mRNA levels of human-specific CYCLOPHILIN over mouse-specific Cyclophilin and showed a significant increase in the FOXP2 knockdown group (**Figure 5C**). Compared to the controls, the shFOXP2 cells produced obvious tumors in the lung tissue of day 60 groups with H&E staining (**Figure 5D**). To analyze the levels of selected genes from the grafted human cancer cells, we designed species-specific qPCR primers for FOXP2, E-cadherin, and PHF2 of human and mouse. Using the total RNAs of lung tissues of day 60 groups, we found that there was no significant difference in the expression of endogenous mouse FOXP2, E-cadherin, and PHF2 between the two groups (**Figure S10**). At the condition that the tail vein-injected shFOXP2 cells produced more tumors in lung tissues, we observed lower mRNA levels of exogenous human FOXP2, E-cadherin, and PHF2 in the harvested samples of the shROXP2 groups than that of the control groups (**Figure 5E**), suggesting that the knockdown of FOXP2 and following down-regulated levels of E-cadherin and PHF2 resulted in elevated abilities of metastasis of the cancer cells. Together, these results determined that inhibition of FOXP2 could enhance the metastasis of breast cancer cells *in vivo*. This was supported by the further analysis of the clinical relevance of FOXP2 expression from TCGA data. The levels of FOXP2 were significantly down-regulated in the group of invasive breast carcinomas compared to normal breast tissue (**Figure 6A**). To further analyze the effect of FOXP2 on the survival of breast cancer patients and test whether its effect relied on the levels of FOXA2, we built two FOXA2-related subgroups of clinical samples, in which the FOXA2^High^ subgroup (n=131) or the FOXA2^Low^ subgroup (n=131) contained either the top 1/3 or the bottom 1/3 of the collected BRCA data set (n=394) respectively according to the levels of FOXA2 expression. In the FOXA2^High^ subgroup, we found that the patients with high levels of FOXP2 showed a better performance on the survival than the patients with low levels of FOXP2 (p=0.03) (**Figure 6B**). However, no significant difference in the survival probability was found between the patients with high or low levels of FOXP2 in the FOXA2^Low^ subgroup (**Figure 6B**). The results confirmed that the FOXP2-improved survival in breast cancer patients was correlated with the high levels of FOXA2.

## Discussion

EMT plays an important role in breast cancer metastasis and in addition, activation of an EMT program enables normal and neoplastic epithelial cells to acquire stem like properties. Multiple members of Fox transcription factor family participate in the regulation of EMT of breast cancer cells. For examples, FOXC1 (43), FOXC2 (44), and FOXM1 (42) are associated with the highly aggressive basal subtype of breast cancers and promote EMT and metastasis. On the other hand, FOXP3 maintains normal epithelial characteristics of breast by selectively inhibiting ZEB2 expression (45). Our previous research found that FOXA2 inhibits EMT in breast cancer cells by stimulating E-cadherin transcription and repressing ZEB2 transcription (11). In this study, FOXA2-interacting FOXP2 was confirmed to act as an EMT suppressor during metastasis in breast cancer cells, adding a new Fox transcription factor to the list of EMT regulators and also providing a further support of FOXA2’s roles on EMT inhibition.

Most studies revealed the predominant repressor role of FOXP2 on the transcription of target genes through interacting with co-repressors such as CtBP-1 (16, 17). For example, FOXP2 was found to bind directly to the promoters of SRPX2 and uPAR, yielding a marked inhibition of their promoter activity (18). Both SRPX2 and uPAR were identified as the stimulators of EMT progression in multiple types of cancers including breast cancer (46–49), providing a clue to explain the role of FOXP2 as an EMT repressor (**Figure 6C**). On the other hand, FOXP2 was also shown to activate gene transcription (14, 20), even though its mechanism was not well described. In this study, we found that FOXP2 could bind to certain promoters and stimulate the transcription of its target genes such as PHF2 and E-cadherin in breast cancer cells (**Figure 6C**). FOXP2 was found to directly bind to and activate the promoter of PHF2 that stimulated the epithelial phenotype of cancer cells (see **Figure 3**). We also provided evidence that the transcriptional activation by FOXP2 could be mediated by FOXA2 at the promoter of E-cadherin. FOXP2, through its zinc-finger/leucine zipper motif (197-408aa), interacted with the DNA binding domain of FOXA2 (166-324aa) (see **Figure 1**) but not the FOXA2 N-terminal and C-terminal transcriptional activation domains that stimulated the transcription of target genes (50). The analysis of clinical samples showed that the FOXP2-improved survival in patients depended on the high expression of FOXA2 in breast cancer (see **Figure 6B**), implicating involvement of FOXA2 for FOXP2 performing its functions. Together, we proposed that FOXP2 could act as a transcriptional activator through its interaction with FOXA2 in breast cancer cells. Furthermore, FOXP2’s zinc-finger/leucine zipper motif was close to its transcriptional repressor region (422-426aa) that mediated the recruitment of its co-repressor CtBP-1 (16, 51). We wondered whether FOXA2 binding to FOXP2’s zinc-finger/leucine zipper motif might prevent the interaction between FOXP2 and CtBP-1 and consequently limit the transcription-repressing abilities of FOXP2. This assumption needs to be investigated in the future.

The expression of FOXP2 itself was also regulated in cancer cells. In this study, we noticed that the expression of FOXP2 was down-regulated during the EGF-induced EMT process (see **Figure 2**) and up-regulated during the Cholera Toxin-induced MET process (see **Figure 3**). Even though dissecting the regulatory mechanisms of FOXP2 expression was out of the range of the current study, some published literatures provided clues for explaining the variation of FOXP2 levels during EMT or MET process. One of the FOXP2 down-regulation mechanisms during the EGF-induced EMT might be mediated by the key mesenchymal transcription factor TWIST (10), whose levels were stimulated by EGF treatment in cancer cells (52). TWIST was found to increase the levels of multiple microRNAs such as the miR-199a-214 cluster, which in turn converged on and repressed the expression of FOXP2 (26, 32). This provided at least one possible mechanism of FOXP2 down-regulation in EGF-induced EMT progression in the cancer cells. The decrease of TWIST levels during the MET progression (38) might also explain the up-regulation of FOXP2 in MET of the Cholera Toxin-induced cancer cells. Furthermore, there was a bona fide binding site on the promoter of FOXP2 for the tumor suppressor p53 (25), whose expression increased in MET (53), implicating that the transcription of FOXP2 in MET might be directly stimulated by p53. Interestingly, PHF2, which was confirmed to be stimulated by FOXP2 in this study, could act as a co-activator of p53 *via* the demethylation of histone H3K9m3 at the promoters of p53 target genes (39), providing a possible positive feedback loop for the up-regulation of FOXP2 during the MET progression in the cancer cells. After all, detailed regulatory mechanisms of FOXP2 expression were worthy to be further studied.

FOXP2 expressed in multiple adult tissues (23, 24) and might act as either a tumor-suppressor or a tumor-stimulator depending on the type of cancers studied (25), presenting a challenge for targeting FOXP2 as a universal target in cancer therapy. This study provided evidence that FOXP2 activated the transcription of its target genes in the maintenance of epithelial characteristics for breast cancer cells. The *in vivo* data showed that the FOXP2-knockdown resulted in the gain of mesenchymal traits in epithelial MCF-7 cells and elevated the migration capabilities of the cells in blood circulation. FOXP2 therefore might be identified as a suppressor of breast cancer metastasis. Interestingly, as a protein interacting with FOXA2, FOXP2 showed the highest correlation with FOXA2 in the basal subtype (see **Figures 1H, I**), which was the most lethal subtype with a high degree of metastasis ability associated with mesenchymal characteristics (54), implicating that the two proteins played important roles together in this subtype of breast cancer. In previous studies, we confirmed that FOXA2 abolished the mesenchymal traits of basal-like MDA-MB-231 cells by stimulating E-cadherin and repressing ZEB2 through recruiting a transcriptional co-repressor TLE3 to ZEB2 promoter (11). In this study, we found that FOXP2 and FOXA2 together synergistically activated the transcription of E-cadherin in MDA-MB-231 cells and resulted in the cells losing their mesenchymal morphology. However, the overexpression of FOXP2 in MDA-MB-231 cells did not affected either the expression of ZEB2 or the FOXA2-mediated repression of ZEB2 promoter (**Figure S11**), suggesting that various mechanisms existed for FOXP2 and FOXA2 to regulate the transcription of their different target genes. The future analysis of RNA-seq and ChIP-seq of the two factors compared in different subtype cells of breast cancer would provide a foundation to describe the detailed picture of FOXP2 and FOXA2 on regulating EMT progression and metastasis of breast cancer cells. Together, this study confirmed that in concert with FOXA2, FOXP2 acted as a tumor-suppressor through inhibiting EMT of breast cancer cells.

## Data Availability Statement

The raw data supporting the conclusions of this article will be made available by the authors, without undue reservation.

## Ethics Statement

The human tissues were obtained and studied in strict adherence to the protocol approved by the Hunan University College of Biology Review Board. The animal study was reviewed and approved by Laboratory Animal Center of Hunan, China (protocol no. SYXK [Xiang] 2008-0001).

## Author Contributions

YXL: conceptualization, methodology, investigation, formal analysis, writing-original draft, writing-review, and editing. TC: conceptualization, methodology, investigation, resources, and formal analysis. MG: resources and investigation. YL: resources and investigation. QZ: resources. GT: methodology and data curation. LY: methodology, data curation, writing-original draft, and project administration. YT: conceptualization, formal analysis, supervision, writing-original draft, writing-review and editing, and project administration. All authors contributed to the article and approved the submitted version.

## Funding

This work was supported by the National Natural Science Foundation of China (grant numbers 81773169 to YT, 81472718 to YT, 31701245 to GT) and the China Changsha Development and Reform Commission “Mass entrepreneurship and innovation program” (2018-68) and “Innovation platform construction program” (2018-216).

## Conflict of Interest

The authors declare that the research was conducted in the absence of any commercial or financial relationships that could be construed as a potential conflict of interest.
