# Supplementary material for: FOXA2-Interacting FOXP2 Prevents Epithelial-Mesenchymal Transition of Breast Cancer Cells by Stimulating E-Cadherin and PHF2 Transcription
Source: Front Oncol. 2021 Feb 25;11:605025. doi: 10.3389/fonc.2021.605025 (PMC7947682; doi:10.3389/fonc.2021.605025)
Supplement: Supplementary file 1 [file DataSheet_1.pdf]

Suppl. Table: TCGA_BRCA_data_set_FOXA2_FOXP2

TCGA_id	fustat	futime	gender	age	reoccur	type	histype	subgroup	sample	FOXA2	FOXP2
TCGA-3C-AAAU	0	3928	female	55	0	infiltrating lobular carcinoma	0	LumB	Tumor	6.44844218	6.724500402
TCGA-5T-A9QA	0	303	female	52	0	other specify	0	Her2	Tumor	6.86449188	7.605259407
TCGA-A1-A0SK	1	967	female	54	0	other specify	0	Basal	Tumor	9.38528446	11.61535111
TCGA-A2-A04T	0	2246	female	62	0	infiltrating ductal carcinoma	0	Basal	Tumor	6.816977221	8.013170581
TCGA-A2-A04U	0	1486	female	47	0	infiltrating ductal carcinoma	0	Basal	Tumor	6.83273544	6.763776804
TCGA-A2-A04V	1	1920	female	39	0	infiltrating ductal carcinoma	0	LumB	Tumor	6.345576482	7.620693707
TCGA-A2-A04W	0	1948	female	50	0	other specify	0	Her2	Tumor	6.382344245	8.014214383
TCGA-A2-A04X	0	1686	female	34	0	infiltrating ductal carcinoma	0	Her2	Tumor	6.331094907	6.829338046
TCGA-A2-A04Y	0	1099	female	53	0	infiltrating ductal carcinoma	0	LumB	Tumor	6.320052725	7.492273197
TCGA-A2-A0CQ	0	2695	female	62	0	infiltrating ductal carcinoma	0	LumA	Tumor	6.330632671	6.808160863
TCGA-A2-A0CT	0	2289	female	71	0	infiltrating ductal carcinoma	0	LumB	Tumor	6.541339144	9.399134132
TCGA-A2-A0CW	0	1948	female	67	0	infiltrating ductal carcinoma	0	LumB	Tumor	6.321568318	6.713957581
TCGA-A2-A0CX	0	1728	female	52	0	infiltrating ductal carcinoma	0	Her2	Tumor	6.347793753	7.459114716
TCGA-A2-A0CY	0	1673	female	63	0	infiltrating ductal carcinoma	0	Her2	Tumor	6.388614374	7.107803109
TCGA-A2-A0CZ	0	1616	female	46	0	infiltrating ductal carcinoma	0	Normal	Tumor	6.435003076	8.804290102
TCGA-A2-A0D1	0	1051	female	76	0	infiltrating ductal carcinoma	0	Her2	Tumor	8.47446773	6.642647514
TCGA-A2-A0D4	0	767	female	37	0	infiltrating ductal carcinoma	0	LumB	Tumor	6.375645673	7.454023124
TCGA-A2-A0EQ	0	2426	female	64	0	infiltrating ductal carcinoma	0	Her2	Tumor	6.693198658	6.944951662
TCGA-A2-A0EV	0	968	female	80	0	infiltrating ductal carcinoma	0	LumB	Tumor	6.398514296	7.610398852
TCGA-A2-A0ST	0	3017	female	62	0	infiltrating ductal carcinoma	0	Basal	Tumor	6.425218807	7.230711149
TCGA-A2-A0SU	0	1662	female	66	0	infiltrating ductal carcinoma	0	LumA	Tumor	6.35297138	7.817439449
TCGA-A2-A0SW	1	1365	female	82	0	infiltrating ductal carcinoma	0	LumB	Tumor	6.426066772	7.681377074
TCGA-A2-A0SX	0	1288	female	48	no	infiltrating ductal carcinoma	0	Basal	Tumor	6.650682193	9.444538925
TCGA-A2-A0SY	0	1347	female	62	0	infiltrating lobular carcinoma	0	LumA	Tumor	6.332295471	8.144021334
TCGA-A2-A0T3	0	569	female	37	0	infiltrating ductal carcinoma	0	LumB	Tumor	6.327102933	8.164604518
TCGA-A2-A0YH	0	659	female	53	0	infiltrating ductal carcinoma	0	LumB	Tumor	6.404553782	7.093890793
TCGA-A2-A0YL	0	445	female	48	0	infiltrating lobular carcinoma	0	Normal	Tumor	6.3233555	8.445642799
TCGA-A2-A0YM	0	965	female	67	0	infiltrating ductal carcinoma	0	Basal	Tumor	7.072052763	7.65357788
TCGA-A2-A1FV	0	714	female	74	0	infiltrating lobular carcinoma	0	LumA	Tumor	6.320232792	8.399847791
TCGA-A2-A1FW	0	528	female	62	0	infiltrating ductal carcinoma	0	LumB	Tumor	6.38641912	8.069148524
TCGA-A2-A1FX	0	1119	female	61	0	infiltrating ductal carcinoma	0	LumB	Tumor	6.3093906	7.424655486
TCGA-A2-A25C	0	523	female	50	0	infiltrating ductal carcinoma	0	LumB	Tumor	6.389510486	8.484222222

TCGA-A2-A25E	0	2796	female	34	0	infiltrating ductal carcinoma	0	LumB	Tumor	6.322974317	7.985653159
TCGA-A2-A25F	0	322	female	66	0	infiltrating ductal carcinoma	0	Basal	Tumor	7.297926805	9.315604562
TCGA-A2-A3XS	1	1032	female	62	0	infiltrating ductal carcinoma	0	Basal	Tumor	6.857520559	7.145124338
TCGA-A2-A3XT	0	2525	female	45	0	infiltrating ductal carcinoma	0	Basal	Tumor	7.977959276	7.336266771
TCGA-A2-A3XU	1	912	female	35	0	infiltrating ductal carcinoma	0	Basal	Tumor	6.552848634	8.696240166
TCGA-A2-A3XV	0	699	female	46	0	infiltrating ductal carcinoma	0	Her2	Tumor	6.528835024	7.948283852
TCGA-A2-A3XY	0	1064	female	49	0	infiltrating ductal carcinoma	0	Basal	Tumor	6.409229258	8.056719233
TCGA-A2-A4RX	0	742	female	67	0	metaplastic carcinoma	0	Basal	Tumor	6.767347811	8.114701705
TCGA-A2-A4S0	0	706	female	77	0	mucinous carcinoma	0	LumA	Tumor	6.376216033	6.817586812
TCGA-A2-A4S3	0	666	female	59	0	infiltrating ductal carcinoma	0	Her2	Tumor	6.373261031	7.373108093
TCGA-A7-A0CD	0	372	female	66	0	infiltrating ductal carcinoma	0	LumA	Tumor	6.382426371	7.116219875
TCGA-A7-A0CG	0	231	female	78	0	nixed histology (please specify)	0	LumA	Tumor	6.616825301	8.372353782
TCGA-A7-A0CH	0	233	female	79	0	infiltrating ductal carcinoma	0	LumA	Tumor	7.103887674	7.886700575
TCGA-A7-A0CH	0	233	female	79	0	infiltrating ductal carcinoma	0	Normal	Normal	6.841095976	9.090400364
TCGA-A7-A0D9	0	273	female	37	0	infiltrating ductal carcinoma	0	Normal	Normal	6.52164009	10.1932104
TCGA-A7-A0D9	0	273	female	37	0	infiltrating ductal carcinoma	0	LumA	Tumor	6.342673293	7.562156305
TCGA-A7-A0DA	0	373	female	62	0	infiltrating ductal carcinoma	0	Basal	Tumor	6.449263207	7.326034913
TCGA-A7-A0DB	0	267	female	56	0	infiltrating ductal carcinoma	0	LumA	Tumor	6.458387921	8.204539974
TCGA-A7-A0DC	0	386	female	63	0	infiltrating ductal carcinoma	0	LumA	Tumor	6.353112207	7.017826153
TCGA-A7-A13D	0	267	female	46	0	infiltrating ductal carcinoma	0	Basal	Tumor	7.021550502	10.26725825
TCGA-A7-A13D	0	267	female	46	0	infiltrating ductal carcinoma	0	Basal	Tumor	6.464557876	7.876536795
TCGA-A7-A13E	0	326	female	62	yes	infiltrating ductal carcinoma	0	Basal	Tumor	6.904433501	9.085236747
TCGA-A7-A13E	0	326	female	62	yes	infiltrating ductal carcinoma	0	Basal	Tumor	6.353018427	7.185690034
TCGA-A7-A13G	0	348	female	79	0	infiltrating ductal carcinoma	0	LumA	Tumor	6.373174878	8.539882371
TCGA-A7-A13H	0	740	female	61	0	infiltrating lobular carcinoma	0	LumA	Tumor	6.322672724	7.725984669
TCGA-A7-A26J	0	369	female	49	0	infiltrating ductal carcinoma	0	LumB	Tumor	6.311900665	7.731574625
TCGA-A7-A3RF	0	408	female	79	0	mucinous carcinoma	0	LumB	Tumor	6.337387073	6.611668508
TCGA-A7-A4SE	0	644	female	54	0	infiltrating ductal carcinoma	0	Basal	Tumor	6.377520713	8.004699394
TCGA-A7-A56D	0	448	female	84	0	infiltrating ductal carcinoma	0	Her2	Tumor	6.419746366	7.664739574
TCGA-A8-A06R	0	547	female	69	0	infiltrating ductal carcinoma	0	LumB	Tumor	6.456054846	7.745571785
TCGA-A8-A06T	0	1614	female	75	0	infiltrating ductal carcinoma	0	LumB	Tumor	6.368408876	7.699445482
TCGA-A8-A06X	1	943	female	77	0	infiltrating ductal carcinoma	0	LumB	Tumor	6.371182633	6.888944875
TCGA-A8-A06Y	0	791	female	66	0	infiltrating ductal carcinoma	0	LumA	Tumor	6.481267329	8.081092643
TCGA-A8-A075	0	518	female	42	0	infiltrating ductal carcinoma	0	Her2	Tumor	6.404975536	7.647992805
TCGA-A8-A076	0	1642	female	66	0	infiltrating ductal carcinoma	0	Her2	Tumor	6.380839082	7.819987446

TCGA-A8-A07B	0	1308	female	69	0	infiltrating ductal carcinoma	0	Her2	Tumor	7.598957251	7.655817608
TCGA-A8-A07E	0	608	female	81	0	infiltrating ductal carcinoma	0	Basal	Tumor	6.316009706	8.417395091
TCGA-A8-A07F	0	577	female	65	0	infiltrating ductal carcinoma	0	Her2	Tumor	6.492303909	9.866998863
TCGA-A8-A07G	0	577	female	65	0	infiltrating ductal carcinoma	0	LumA	Tumor	6.328059891	8.448533601
TCGA-A8-A07O	0	304	female	51	0	infiltrating ductal carcinoma	0	Basal	Tumor	6.436187849	9.307816615
TCGA-A8-A07P	0	334	female	68	0	infiltrating ductal carcinoma	0	LumB	Tumor	6.336460859	7.704311766
TCGA-A8-A07S	0	243	female	73	0	nixed histology (please specify)	0	LumB	Tumor	6.400126564	7.192134275
TCGA-A8-A082	0	-31	female	58	0	infiltrating ductal carcinoma	0	LumB	Tumor	6.416971915	7.080493506
TCGA-A8-A084	0	458	female	81	0	infiltrating ductal carcinoma	0	LumB	Tumor	6.343547632	7.19130622
TCGA-A8-A086	0	396	female	59	0	infiltrating ductal carcinoma	0	LumB	Tumor	6.323102842	7.298564369
TCGA-A8-A08B	0	1156	female	52	0	infiltrating ductal carcinoma	0	Her2	Tumor	8.079299926	7.365409389
TCGA-A8-A08C	0	881	female	65	0	infiltrating ductal carcinoma	0	LumA	Tumor	6.571039895	7.223769359
TCGA-A8-A08G	0	607	female	41	0	infiltrating ductal carcinoma	0	LumB	Tumor	6.456213009	7.210788838
TCGA-A8-A08I	0	365	female	53	0	infiltrating ductal carcinoma	0	LumB	Tumor	6.345691569	6.887945432
TCGA-A8-A08L	1	304	female	89	0	infiltrating ductal carcinoma	0	Her2	Tumor	6.593831098	6.969729288
TCGA-A8-A08O	0	943	female	45	0	infiltrating ductal carcinoma	0	LumA	Tumor	6.347372765	8.076782967
TCGA-A8-A08P	0	943	female	70	0	infiltrating ductal carcinoma	0	LumB	Tumor	7.05399111	7.467016123
TCGA-A8-A08S	0	1004	female	71	0	infiltrating ductal carcinoma	0	LumB	Tumor	6.327282972	7.215248262
TCGA-A8-A08X	0	1308	female	43	0	infiltrating ductal carcinoma	0	Her2	Tumor	6.67899547	7.507403745
TCGA-A8-A091	0	1004	female	61	0	infiltrating ductal carcinoma	0	LumA	Tumor	6.360009625	7.887142612
TCGA-A8-A092	0	942	female	48	0	infiltrating ductal carcinoma	0	Her2	Tumor	6.430386749	6.943271827
TCGA-A8-A095	0	1277	female	45	0	infiltrating ductal carcinoma	0	LumB	Tumor	6.917791964	7.687136588
TCGA-A8-A099	0	304	female	76	0	infiltrating ductal carcinoma	0	LumA	Tumor	6.321817911	7.280139171
TCGA-A8-A09E	0	1492	female	73	0	infiltrating ductal carcinoma	0	LumB	Tumor	6.346330139	7.149941772
TCGA-A8-A09K	0	912	female	68	0	infiltrating ductal carcinoma	0	LumB	Tumor	6.422381891	7.115720503
TCGA-A8-A09M	0	1006	female	75	0	infiltrating ductal carcinoma	0	LumB	Tumor	6.404347127	7.004635344
TCGA-A8-A09Q	0	761	female	83	0	infiltrating ductal carcinoma	0	LumB	Tumor	6.327387666	8.547883247
TCGA-A8-A09R	0	273	female	82	0	infiltrating ductal carcinoma	0	LumB	Tumor	6.425931056	7.565415809
TCGA-A8-A09T	0	579	female	68	0	infiltrating lobular carcinoma	0	LumB	Tumor	7.460950973	7.373772663
TCGA-A8-A0A4	0	396	female	73	0	infiltrating lobular carcinoma	0	Normal	Tumor	6.416128314	8.640969946
TCGA-A8-A0A6	0	640	female	64	0	infiltrating lobular carcinoma	0	LumA	Tumor	6.367828002	7.231081409
TCGA-A8-A0AD	0	1157	female	83	0	nixed histology (please specify)	0	LumA	Tumor	6.356715945	7.982714428
TCGA-AC-A2B8	0	677	female	84	0	infiltrating lobular carcinoma	0	LumA	Tumor	6.305465013	8.352377722
TCGA-AC-A2FK	0	2650	female	45	0	infiltrating lobular carcinoma	0	Normal	Tumor	6.346444139	8.52184482
TCGA-AC-A2QH	0	1005	female	58	0	metaplastic carcinoma	0	Basal	Tumor	7.113509322	12.81178497

TCGA-AC-A2QI	0	588	female	76	0	infiltrating lobular carcinoma	0	LumA	Tumor	6.324528824	7.851264845
TCGA-AC-A3W5	0	504	female	65	0	infiltrating lobular carcinoma	0	Her2	Tumor	6.412592061	7.807649956
TCGA-AC-A3W7	0	471	female	66	0	infiltrating lobular carcinoma	0	LumA	Tumor	6.661211076	7.798811935
TCGA-AC-A6IV	0	568	female	47	0	infiltrating lobular carcinoma	0	Normal	Tumor	6.488892419	8.228635929
TCGA-AC-A6IW	0	413	female	73	0	infiltrating ductal carcinoma	0	Basal	Tumor	6.327713512	7.049710039
TCGA-AC-A6IX	0	373	female	49	0	infiltrating lobular carcinoma	0	LumA	Tumor	6.449757138	8.116774084
TCGA-AC-A6IX	0	373	female	49	0	infiltrating lobular carcinoma	0	LumA	Tumor	6.314951278	7.610961368
TCGA-AC-A7VB	0	250	female	51	0	infiltrating ductal carcinoma	0	LumB	Tumor	6.318511349	7.508703829
TCGA-AN-A03X	0	10	female	74	0	infiltrating ductal carcinoma	0	LumB	Tumor	6.399989037	8.633728592
TCGA-AN-A03Y	0	10	female	66	0	infiltrating ductal carcinoma	0	LumB	Tumor	6.337566559	7.378824679
TCGA-AN-A041	0	7	female	29	0	infiltrating ductal carcinoma	0	LumB	Tumor	6.328617435	7.456009495
TCGA-AN-A046	0	10	female	68	0	infiltrating ductal carcinoma	0	LumB	Tumor	6.33788503	7.738893349
TCGA-AN-A049	0	19	female	62	0	infiltrating ductal carcinoma	0	LumB	Tumor	6.424220644	7.60595105
TCGA-AN-A04A	0	90	female	36	0	infiltrating ductal carcinoma	0	Normal	Tumor	6.335293795	7.937521602
TCGA-AN-A04C	0	54	female	51	0	infiltrating ductal carcinoma	0	Her2	Tumor	6.4067673	6.964160791
TCGA-AN-A04D	0	52	female	58	0	infiltrating ductal carcinoma	0	Basal	Tumor	7.38999694	6.56887884
TCGA-AN-A0AJ	0	303	female	79	0	infiltrating ductal carcinoma	0	LumB	Tumor	6.568963577	7.206749297
TCGA-AN-A0AK	0	224	female	76	0	infiltrating ductal carcinoma	0	LumB	Tumor	6.353688127	6.961816768
TCGA-AN-A0AL	0	227	female	41	0	infiltrating ductal carcinoma	0	Basal	Tumor	6.477647573	7.469711853
TCGA-AN-A0AM	0	5	female	56	0	infiltrating ductal carcinoma	0	Her2	Tumor	6.728150591	7.389438717
TCGA-AN-A0AR	0	10	female	55	0	infiltrating ductal carcinoma	0	Basal	Tumor	6.541487462	6.975824075
TCGA-AN-A0AS	0	10	female	70	0	infiltrating ductal carcinoma	0	LumB	Tumor	7.104964151	8.774327091
TCGA-AN-A0FK	0	213	female	88	0	infiltrating ductal carcinoma	0	LumB	Tumor	6.344906538	7.049419194
TCGA-AN-A0FS	0	210	female	55	0	infiltrating ductal carcinoma	0	LumA	Tumor	6.362850054	8.230391739
TCGA-AN-A0FW	0	11	female	67	0	infiltrating ductal carcinoma	0	LumB	Tumor	6.388085985	7.600254417
TCGA-AN-A0FX	0	10	female	52	0	infiltrating ductal carcinoma	0	Basal	Tumor	6.67690941	9.082349714
TCGA-AN-A0FY	0	10	female	55	0	infiltrating ductal carcinoma	0	LumB	Tumor	6.311814393	7.563564234
TCGA-AN-A0G0	0	16	female	56	0	infiltrating ductal carcinoma	0	Basal	Tumor	8.330637675	7.358976897
TCGA-AN-A0XN	0	10	female	68	0	infiltrating ductal carcinoma	0	Her2	Tumor	6.367061148	9.473332688
TCGA-AN-A0XS	0	10	female	63	0	infiltrating ductal carcinoma	0	LumA	Tumor	6.327360493	8.172028123
TCGA-AN-A0XU	0	10	female	54	0	infiltrating ductal carcinoma	0	Basal	Tumor	6.618570697	7.07022319
TCGA-AO-A03N	0	1645	female	59	yes	infiltrating ductal carcinoma	0	Her2	Tumor	6.707413243	8.129607806
TCGA-AO-A03O	1	2483	female	69	0	infiltrating ductal carcinoma	0	LumB	Tumor	6.789838759	7.273044731
TCGA-AO-A03R	0	1707	female	57	no	infiltrating ductal carcinoma	0	Basal	Tumor	6.989091457	7.248920565
TCGA-AO-A03V	0	886	female	41	no	infiltrating ductal carcinoma	0	LumA	Tumor	6.341065324	7.780954071

TCGA-AO-A0J4	0	294	female	41	no	infiltrating ductal carcinoma	0	Basal	Tumor	7.660257942	8.124971715
TCGA-AO-A0JB	0	1150	female	50	no	infiltrating ductal carcinoma	0	Basal	Tumor	7.082084368	7.866502534
TCGA-AO-A0JC	0	1547	female	64	no	infiltrating ductal carcinoma	0	Basal	Tumor	6.418963503	7.853958482
TCGA-AO-A0JD	0	1813	female	59	no	infiltrating ductal carcinoma	0	Basal	Tumor	6.345127757	7.503042438
TCGA-AO-A0JE	0	1966	female	53	no	infiltrating ductal carcinoma	0	Her2	Tumor	6.34321141	7.879317236
TCGA-AO-A0JI	0	1172	female	56	no	infiltrating ductal carcinoma	0	LumA	Tumor	6.343791501	8.871321726
TCGA-AO-A0JJ	0	1512	female	54	no	infiltrating lobular carcinoma	0	LumA	Tumor	6.402764259	9.145968508
TCGA-AO-A125	0	3019	female	72	no	other specify	0	LumA	Tumor	6.553413373	6.169827717
TCGA-AO-A1KS	0	16	female	69	no	infiltrating lobular carcinoma	0	LumB	Tumor	6.388227746	7.77851742
TCGA-AQ-A1H3	0	402	female	49	0	infiltrating ductal carcinoma	0	LumA	Tumor	6.312313029	8.197752019
TCGA-AR-A0TP	0	2521	female	43	0	infiltrating ductal carcinoma	0	Basal	Tumor	7.269139888	6.874995864
TCGA-AR-A0TR	1	160	female	68	0	infiltrating ductal carcinoma	0	LumA	Tumor	6.315239571	6.650087844
TCGA-AR-A0TS	0	1138	female	46	0	infiltrating ductal carcinoma	0	Basal	Tumor	7.441495527	7.545524599
TCGA-AR-A0U0	0	1988	female	73	0	infiltrating ductal carcinoma	0	Basal	Tumor	6.346489589	6.475434559
TCGA-AR-A0U2	1	2551	female	47	0	infiltrating ductal carcinoma	0	Her2	Tumor	6.518559992	7.30012886
TCGA-AR-A0U4	0	1684	female	54	0	infiltrating ductal carcinoma	0	Basal	Tumor	6.504102543	7.740844737
TCGA-AR-A1AN	0	1330	female	46	0	infiltrating ductal carcinoma	0	LumA	Tumor	6.333420745	8.376208821
TCGA-AR-A1AR	1	524	female	50	yes	infiltrating ductal carcinoma	0	Basal	Tumor	6.30691982	8.141746183
TCGA-AR-A1AS	0	1150	female	54	0	infiltrating ductal carcinoma	0	LumB	Tumor	6.360408269	7.343414567
TCGA-AR-A1AW	0	1072	female	65	0	infiltrating ductal carcinoma	0	LumB	Tumor	6.335892913	7.62960588
TCGA-AR-A1AX	0	1103	female	64	0	infiltrating ductal carcinoma	0	Basal	Tumor	6.334438271	7.617784634
TCGA-AR-A1AY	0	1026	female	65	0	infiltrating ductal carcinoma	0	Basal	Tumor	6.583664713	8.913414362
TCGA-AR-A24M	0	1991	female	38	0	nixed histology (please specify)	0	LumA	Tumor	6.367544881	8.335228412
TCGA-AR-A24N	0	2074	female	54	0	infiltrating ductal carcinoma	0	LumB	Tumor	6.319818788	8.097133128
TCGA-AR-A24Q	0	3088	female	49	0	infiltrating ductal carcinoma	0	Basal	Tumor	7.533648017	8.723969185
TCGA-AR-A251	0	1374	female	51	0	infiltrating ductal carcinoma	0	Basal	Tumor	7.375806412	7.639594343
TCGA-AR-A256	1	2854	female	45	0	infiltrating ductal carcinoma	0	Basal	Tumor	6.326118351	7.027745849
TCGA-AR-A2LM	0	1585	female	49	0	infiltrating lobular carcinoma	0	LumA	Tumor	6.376017014	8.146259669
TCGA-AR-A2LR	0	846	female	49	0	metaplastic carcinoma	0	Basal	Tumor	8.7031816	9.622393241
TCGA-AR-A5QP	0	622	female	54	0	infiltrating lobular carcinoma	0	Normal	Tumor	6.340637218	8.585343646
TCGA-AR-A5QQ	1	322	female	68	0	other specify	0	Basal	Tumor	7.35287323	7.006524622
TCGA-B6-A0I1	1	2361	female	73	0	infiltrating ductal carcinoma	0	Basal	Tumor	6.394270484	6.588672745
TCGA-B6-A0I6	1	991	female	49	0	infiltrating ductal carcinoma	0	Basal	Tumor	6.431637569	7.439318689
TCGA-B6-A0I9	1	362	female	62	0	infiltrating ductal carcinoma	0	Her2	Tumor	6.327438041	6.814615049
TCGA-B6-A0IC	1	1542	female	90	0	other specify	0	LumB	Tumor	6.30504968	6.169827717

TCGA-B6-A0IE	1	1993	female	38	0	nixed histology (please specify)	0	Basal	Tumor	6.453806756	7.918701257
TCGA-B6-A0IH	1	3418	female	81	0	infiltrating lobular carcinoma	0	LumA	Tumor	6.361896484	8.027128084
TCGA-B6-A0IO	0	3350	female	66	0	infiltrating ductal carcinoma	0	LumB	Tumor	6.324628241	7.219523831
TCGA-B6-A0RG	0	2082	female	26	0	infiltrating ductal carcinoma	0	LumA	Tumor	6.575144267	7.663929228
TCGA-B6-A0RL	1	2469	female	60	0	infiltrating ductal carcinoma	0	LumB	Tumor	6.320896541	9.757750333
TCGA-B6-A0RM	1	2373	female	57	0	infiltrating ductal carcinoma	0	LumB	Tumor	6.457843903	7.916028704
TCGA-B6-A0RQ	1	4267	female	68	0	infiltrating lobular carcinoma	0	Normal	Tumor	6.572048901	7.487691694
TCGA-B6-A0RT	0	2721	female	39	0	infiltrating ductal carcinoma	0	Basal	Tumor	6.474654006	7.939372079
TCGA-B6-A0WZ	0	3941	female	50	0	infiltrating ductal carcinoma	0	LumB	Tumor	6.410620022	7.407185066
TCGA-B6-A0X1	0	5677	female	48	0	infiltrating ductal carcinoma	0	Basal	Tumor	6.580927312	7.695332831
TCGA-B6-A1KF	0	3088	female	68	0	infiltrating ductal carcinoma	0	Basal	Tumor	6.367960757	6.792038409
TCGA-B6-A408	0	2072	female	55	0	nixed histology (please specify)	0	LumA	Tumor	6.32695181	7.586859019
TCGA-B6-A40B	0	3152	female	76	0	infiltrating lobular carcinoma	0	LumA	Tumor	6.327839096	7.767457428
TCGA-B6-A40C	0	2164	female	51	0	infiltrating lobular carcinoma	0	Her2	Tumor	6.313229234	7.272407648
TCGA-BH-A0AV	0	1820	female	52	0	infiltrating ductal carcinoma	0	Basal	Tumor	6.83211327	8.790875508
TCGA-BH-A0AY	0	777	female	62	0	infiltrating ductal carcinoma	0	LumB	Tumor	6.48759259	8.068591435
TCGA-BH-A0AZ	0	1919	female	47	0	infiltrating ductal carcinoma	0	Normal	Normal	6.315788884	9.171938297
TCGA-BH-A0AZ	0	1919	female	47	0	infiltrating ductal carcinoma	0	LumA	Tumor	6.361509648	8.464875441
TCGA-BH-A0B3	0	1203	female	53	0	infiltrating ductal carcinoma	0	Normal	Normal	6.38558832	8.941320282
TCGA-BH-A0B7	0	2559	female	42	0	infiltrating ductal carcinoma	0	Her2	Tumor	6.535991637	8.280933472
TCGA-BH-A0B8	0	1569	female	64	0	infiltrating ductal carcinoma	0	LumA	Tumor	6.596397997	7.813290005
TCGA-BH-A0B9	0	1572	female	44	0	infiltrating ductal carcinoma	0	Basal	Tumor	6.45733692	9.607232711
TCGA-BH-A0BA	0	1132	female	51	0	nixed histology (please specify)	0	LumA	Tumor	6.703291963	8.245118456
TCGA-BH-A0BA	0	1132	female	51	0	nixed histology (please specify)	0	Normal	Normal	7.523765979	9.665667784
TCGA-BH-A0BD	0	554	female	47	0	infiltrating ductal carcinoma	0	Basal	Tumor	6.391074983	7.809785446
TCGA-BH-A0BG	0	1871	female	73	0	infiltrating ductal carcinoma	0	Basal	Tumor	6.433002929	7.886292673
TCGA-BH-A0BJ	0	660	female	41	0	infiltrating ductal carcinoma	0	Normal	Normal	6.340331178	8.815780243
TCGA-BH-A0BM	0	1876	female	54	0	infiltrating ductal carcinoma	0	Normal	Normal	6.346923365	7.830817866
TCGA-BH-A0BQ	0	2255	female	39	0	infiltrating ductal carcinoma	0	Normal	Normal	6.346954779	9.11390506
TCGA-BH-A0BT	0	2365	female	56	0	infiltrating ductal carcinoma	0	LumA	Tumor	6.343428794	7.586199735
TCGA-BH-A0BV	0	1519	female	78	0	infiltrating ductal carcinoma	0	Normal	Normal	6.377015149	8.883067004
TCGA-BH-A0BW	0	2371	female	71	0	infiltrating ductal carcinoma	0	Basal	Tumor	6.335853617	7.121123144
TCGA-BH-A0C0	0	1270	female	62	0	infiltrating ductal carcinoma	0	Normal	Normal	6.316995483	8.822454004
TCGA-BH-A0C0	0	1270	female	62	0	infiltrating ductal carcinoma	0	LumB	Tumor	6.433010305	7.186262888
TCGA-BH-A0C7	0	2767	female	48	0	infiltrating ductal carcinoma	0	LumB	Tumor	6.308965975	7.817344933

TCGA-BH-A0DG	0	2041	female	30	0	infiltrating ductal carcinoma	0	Her2	Tumor	6.347398646	8.28508522
TCGA-BH-A0DK	0	423	female	49	0	infiltrating ductal carcinoma	0	Normal	Normal	7.068972492	8.197431141
TCGA-BH-A0DP	0	476	female	60	0	infiltrating lobular carcinoma	0	Normal	Normal	6.392936642	8.060175743
TCGA-BH-A0DQ	0	98	female	42	0	infiltrating ductal carcinoma	0	LumA	Tumor	6.343388746	8.290632475
TCGA-BH-A0DQ	0	98	female	42	0	infiltrating ductal carcinoma	0	Normal	Normal	6.370578948	8.816485474
TCGA-BH-A0DT	0	2403	female	41	0	infiltrating ductal carcinoma	0	Normal	Normal	6.596468349	8.346891236
TCGA-BH-A0DT	0	2403	female	41	0	infiltrating ductal carcinoma	0	LumA	Tumor	6.33839682	7.850212584
TCGA-BH-A0DV	0	2064	female	54	0	infiltrating ductal carcinoma	0	LumA	Tumor	6.324367456	8.843383481
TCGA-BH-A0DV	0	2064	female	54	0	infiltrating ductal carcinoma	0	Normal	Normal	6.818740822	8.350668591
TCGA-BH-A0DZ	0	495	female	43	0	infiltrating ductal carcinoma	0	Normal	Normal	6.617574031	7.659969448
TCGA-BH-A0DZ	0	495	female	43	0	infiltrating ductal carcinoma	0	Her2	Tumor	6.993415405	7.878106653
TCGA-BH-A0E1	0	477	female	52	0	infiltrating ductal carcinoma	0	LumB	Tumor	6.348380279	7.4845448
TCGA-BH-A0E6	0	293	female	69	0	infiltrating ductal carcinoma	0	Basal	Tumor	6.440896624	7.575465105
TCGA-BH-A0EB	0	745	female	69	0	infiltrating ductal carcinoma	0	LumA	Tumor	6.327314532	7.648116966
TCGA-BH-A0GZ	0	328	female	62	0	infiltrating ductal carcinoma	0	LumA	Tumor	6.43354945	7.674227483
TCGA-BH-A0H0	0	461	female	69	0	infiltrating ductal carcinoma	0	LumB	Tumor	6.373280992	7.087396916
TCGA-BH-A0H5	0	1620	female	45	0	infiltrating ductal carcinoma	0	Normal	Normal	6.408792983	9.022591362
TCGA-BH-A0H5	0	1620	female	45	0	infiltrating ductal carcinoma	0	LumA	Tumor	6.339362041	8.23843483
TCGA-BH-A0H7	0	702	female	65	0	infiltrating ductal carcinoma	0	LumB	Tumor	6.331349233	7.964082261
TCGA-BH-A0H7	0	702	female	65	0	infiltrating ductal carcinoma	0	Normal	Normal	6.46266127	8.461536469
TCGA-BH-A0H9	0	1247	female	69	0	infiltrating ductal carcinoma	0	Normal	Normal	6.634254034	9.660415747
TCGA-BH-A0HB	0	806	female	55	0	infiltrating ductal carcinoma	0	LumB	Tumor	6.363577967	7.785494071
TCGA-BH-A0HF	0	727	female	77	0	infiltrating ductal carcinoma	0	LumA	Tumor	6.533384608	8.730619971
TCGA-BH-A0HK	0	178	female	81	0	infiltrating ductal carcinoma	0	LumA	Tumor	6.522958977	8.588131557
TCGA-BH-A0HP	0	414	female	65	0	infiltrating lobular carcinoma	0	Normal	Tumor	6.323838269	7.358887121
TCGA-BH-A0HQ	0	1121	female	56	0	infiltrating ductal carcinoma	0	LumA	Tumor	6.431230223	7.781160201
TCGA-BH-A0HX	0	829	female	54	0	infiltrating ductal carcinoma	0	LumB	Tumor	6.463126608	7.912377564
TCGA-BH-A0W7	0	1363	female	49	0	infiltrating ductal carcinoma	0	Her2	Tumor	6.410684751	7.866734385
TCGA-BH-A0WA	0	701	female	82	0	metaplastic carcinoma	0	Basal	Tumor	7.101171686	8.350253343
TCGA-BH-A202	0	795	female	60	0	infiltrating ductal carcinoma	0	Her2	Tumor	6.369981173	7.848330964
TCGA-BH-A6R8	0	293	female	46	0	infiltrating ductal carcinoma	0	LumA	Tumor	6.334871522	6.761079785
TCGA-BH-A6R9	0	160	female	61	0	metaplastic carcinoma	0	Basal	Tumor	6.608042317	8.215488548
TCGA-BH-A8FZ	0	574	female	58	0	infiltrating lobular carcinoma	0	Normal	Tumor	6.416875048	8.348559874
TCGA-C8-A12M	0	358	female	70	0	infiltrating ductal carcinoma	0	LumB	Tumor	6.373912014	7.949082885
TCGA-C8-A12N	0	358	female	58	0	infiltrating ductal carcinoma	0	LumA	Tumor	6.340204314	8.080007968

TCGA-C8-A12U	0	385	female	46	0	infiltrating ductal carcinoma	0	LumB	Tumor	6.6545916	6.353789753
TCGA-C8-A12W	0	385	female	49	0	infiltrating ductal carcinoma	0	Her2	Tumor	6.899878067	7.647710449
TCGA-C8-A12Z	0	382	female	45	0	infiltrating ductal carcinoma	0	Her2	Tumor	6.368071142	7.4164556
TCGA-C8-A130	0	96	female	52	0	infiltrating ductal carcinoma	0	Her2	Tumor	6.336727352	7.480900092
TCGA-C8-A135	0	32	female	64	0	infiltrating ductal carcinoma	0	Her2	Tumor	6.588971083	8.154364571
TCGA-C8-A1HE	0	375	female	59	0	infiltrating ductal carcinoma	0	LumA	Tumor	6.312764502	7.234521186
TCGA-C8-A1HG	0	345	female	50	0	infiltrating ductal carcinoma	0	LumB	Tumor	6.702588828	7.192720912
TCGA-C8-A1HO	0	375	female	34	0	infiltrating ductal carcinoma	0	LumB	Tumor	6.324023563	7.420960847
TCGA-C8-A26W	0	381	female	58	0	infiltrating ductal carcinoma	0	Her2	Tumor	6.368004512	7.728413641
TCGA-C8-A274	0	290	female	63	0	infiltrating ductal carcinoma	0	LumB	Tumor	6.315739621	6.959467235
TCGA-C8-A27B	0	439	female	48	0	infiltrating ductal carcinoma	0	Basal	Tumor	6.651239606	6.460984391
TCGA-C8-A3M8	0	394	female	68	0	infiltrating lobular carcinoma	0	LumB	Tumor	6.310909684	7.158307282
TCGA-C8-A8HP	0	396	female	59	0	metaplastic carcinoma	0	Her2	Tumor	6.474141439	7.203692029
TCGA-D8-A13Y	0	362	female	52	0	infiltrating ductal carcinoma	0	Her2	Tumor	9.549769082	7.079306056
TCGA-D8-A142	0	425	female	74	0	infiltrating ductal carcinoma	0	Basal	Tumor	8.233864628	10.42143543
TCGA-D8-A146	0	643	female	57	0	infiltrating ductal carcinoma	0	LumA	Tumor	6.327486985	7.908883259
TCGA-D8-A147	0	584	female	45	0	infiltrating ductal carcinoma	0	Basal	Tumor	6.800363284	7.041995036
TCGA-D8-A1J9	0	532	female	48	0	infiltrating ductal carcinoma	0	Her2	Tumor	11.11341595	7.346224006
TCGA-D8-A1JE	0	575	female	62	0	infiltrating ductal carcinoma	0	LumB	Tumor	6.31889698	8.2357001
TCGA-D8-A1JF	0	366	female	79	0	infiltrating ductal carcinoma	0	Her2	Tumor	6.550331101	9.136844647
TCGA-D8-A1JG	0	366	female	62	0	infiltrating ductal carcinoma	0	Her2	Tumor	6.311026814	8.700032319
TCGA-D8-A1JM	0	590	female	59	0	infiltrating ductal carcinoma	0	Basal	Tumor	6.718130138	10.66135987
TCGA-D8-A1JN	0	620	female	80	0	infiltrating lobular carcinoma	0	LumA	Tumor	6.372671324	6.971516046
TCGA-D8-A1JP	0	639	female	73	0	infiltrating ductal carcinoma	0	LumB	Tumor	6.541583923	8.19024577
TCGA-D8-A1JU	0	447	female	51	0	infiltrating ductal carcinoma	0	LumA	Tumor	6.322523506	9.201801659
TCGA-D8-A1XG	0	448	female	86	0	infiltrating ductal carcinoma	0	LumA	Tumor	6.343460958	6.688248136
TCGA-D8-A1XJ	0	664	female	76	no	other specify	0	Her2	Tumor	6.392695659	7.591421677
TCGA-D8-A1XK	0	441	female	55	0	infiltrating ductal carcinoma	0	Basal	Tumor	6.98950992	7.395911719
TCGA-D8-A1XM	0	538	female	57	0	infiltrating ductal carcinoma	0	LumA	Tumor	6.30623842	8.082616214
TCGA-D8-A1XQ	0	499	female	69	0	infiltrating ductal carcinoma	0	Basal	Tumor	6.319576501	7.123298976
TCGA-D8-A1XR	0	482	female	56	0	infiltrating ductal carcinoma	0	LumB	Tumor	6.307200273	7.385534672
TCGA-D8-A1XW	0	385	female	53	0	infiltrating ductal carcinoma	0	Basal	Tumor	6.472264653	7.076351746
TCGA-D8-A1Y0	0	472	female	65	0	infiltrating ductal carcinoma	0	LumB	Tumor	6.300893862	7.934893121
TCGA-D8-A1Y3	0	430	female	61	0	infiltrating ductal carcinoma	0	Her2	Tumor	6.334625176	6.782171791
TCGA-D8-A27E	0	530	female	66	0	other specify	0	LumA	Tumor	6.383200143	6.916538136

TCGA-D8-A27F	0	488	female	40	0	infiltrating ductal carcinoma	0	Basal	Tumor	7.11577321	7.665520647
TCGA-D8-A27H	0	397	female	72	0	infiltrating ductal carcinoma	0	Basal	Tumor	6.747062494	7.877468071
TCGA-D8-A27I	0	439	female	58	0	infiltrating lobular carcinoma	0	Normal	Tumor	6.311748039	8.819743236
TCGA-D8-A27M	0	410	female	59	0	infiltrating ductal carcinoma	0	Basal	Tumor	6.468193647	8.383595839
TCGA-D8-A27N	0	519	female	36	0	infiltrating ductal carcinoma	0	Her2	Tumor	6.325474561	8.037038634
TCGA-D8-A27P	0	49	female	64	0	infiltrating ductal carcinoma	0	LumA	Tumor	6.465733735	7.335820671
TCGA-D8-A27T	0	398	female	53	0	infiltrating lobular carcinoma	0	LumB	Tumor	6.324597472	8.186040785
TCGA-D8-A27W	0	373	female	55	0	mucinous carcinoma	0	LumB	Tumor	6.307467706	7.125869073
TCGA-D8-A73U	0	492	female	88	0	infiltrating lobular carcinoma	0	LumA	Tumor	6.492216795	7.82692383
TCGA-D8-A73W	0	244	female	79	0	mucinous carcinoma	0	LumB	Tumor	6.422060329	7.323431041
TCGA-D8-A73X	0	368	female	53	0	other specify	0	LumA	Tumor	6.331052007	7.822742849
TCGA-E2-A105	0	1308	female	79	0	infiltrating ductal carcinoma	0	LumB	Tumor	6.320783968	7.204675006
TCGA-E2-A10A	0	1229	female	41	yes	infiltrating ductal carcinoma	0	LumB	Tumor	6.324740082	7.177297815
TCGA-E2-A10B	0	1141	female	67	0	infiltrating ductal carcinoma	0	LumB	Tumor	6.316300076	7.860161469
TCGA-E2-A10E	0	865	female	64	0	infiltrating ductal carcinoma	0	LumB	Tumor	6.509337406	7.60145333
TCGA-E2-A10F	0	878	female	47	0	infiltrating lobular carcinoma	0	LumA	Tumor	6.331630759	8.060127963
TCGA-E2-A14Q	0	1163	female	50	0	infiltrating ductal carcinoma	0	LumA	Tumor	6.327946308	7.924738359
TCGA-E2-A14R	0	1174	female	62	0	infiltrating ductal carcinoma	0	Basal	Tumor	6.359845627	6.59350378
TCGA-E2-A14T	0	1013	female	52	0	infiltrating ductal carcinoma	0	LumB	Tumor	6.314577672	7.265037748
TCGA-E2-A14U	0	1318	female	74	0	infiltrating lobular carcinoma	0	Normal	Tumor	6.473119393	7.066859336
TCGA-E2-A14Y	0	870	female	35	0	infiltrating ductal carcinoma	0	Basal	Tumor	6.47304495	7.888951494
TCGA-E2-A153	0	707	female	51	0	infiltrating ductal carcinoma	0	Normal	Normal	7.550554132	8.326037747
TCGA-E2-A154	0	591	female	68	0	infiltrating ductal carcinoma	0	LumB	Tumor	6.362787496	7.328783099
TCGA-E2-A159	0	762	female	50	0	infiltrating ductal carcinoma	0	Basal	Tumor	6.81266128	7.951142914
TCGA-E2-A15E	0	630	female	40	0	infiltrating ductal carcinoma	0	Normal	Tumor	6.638053964	7.799091753
TCGA-E2-A15F	0	658	female	64	0	infiltrating ductal carcinoma	0	Normal	Tumor	6.347422472	7.267105217
TCGA-E2-A15G	0	554	female	76	0	nixed histology (please specify)	0	LumA	Tumor	6.34561253	7.146097812
TCGA-E2-A15I	0	530	female	44	0	infiltrating ductal carcinoma	0	LumA	Tumor	6.330931084	8.031681133
TCGA-E2-A15S	0	428	female	34	0	infiltrating ductal carcinoma	0	LumB	Tumor	6.374370304	7.871614645
TCGA-E2-A1B6	0	867	female	44	0	infiltrating ductal carcinoma	0	Basal	Tumor	6.456826396	7.467825075
TCGA-E2-A1BD	0	520	female	53	0	infiltrating ductal carcinoma	0	LumA	Tumor	6.508409145	7.897478869
TCGA-E2-A1IF	0	1138	female	74	0	infiltrating ductal carcinoma	0	LumA	Tumor	6.416404812	8.120005808
TCGA-E2-A1II	0	1025	female	51	0	infiltrating ductal carcinoma	0	Basal	Tumor	6.372315508	8.839155554
TCGA-E2-A1IJ	0	865	female	57	0	infiltrating lobular carcinoma	0	LumA	Tumor	6.472435448	8.431090098
TCGA-E2-A1L7	0	834	female	40	0	infiltrating ductal carcinoma	0	LumB	Tumor	6.588069447	7.254539705

TCGA-E2-A1LB	0	1231	female	41	0	infiltrating ductal carcinoma	0	Normal	Normal	6.341015838	8.688409064
TCGA-E2-A1LB	0	1231	female	41	0	infiltrating ductal carcinoma	0	Her2	Tumor	6.440912725	7.852973578
TCGA-E2-A1LG	0	574	female	50	0	infiltrating lobular carcinoma	0	Basal	Tumor	6.486467063	9.48483535
TCGA-E2-A1LH	0	3247	female	59	0	infiltrating ductal carcinoma	0	Basal	Tumor	7.92898154	7.656296538
TCGA-E2-A1LI	0	3121	female	57	0	infiltrating ductal carcinoma	0	Basal	Tumor	6.321328316	8.550442143
TCGA-E2-A1LS	0	470	female	46	0	infiltrating ductal carcinoma	0	Normal	Normal	6.34588869	11.58344864
TCGA-E2-A2P6	0	168	female	77	0	infiltrating lobular carcinoma	0	LumB	Tumor	6.326555744	7.199701515
TCGA-E2-A570	0	931	female	47	0	infiltrating ductal carcinoma	0	LumB	Tumor	6.357691907	7.864142491
TCGA-E2-A572	0	1208	female	72	0	infiltrating ductal carcinoma	0	LumA	Tumor	6.396916104	7.008531683
TCGA-E2-A574	0	808	female	44	0	infiltrating ductal carcinoma	0	Basal	Tumor	6.375711664	8.850987287
TCGA-E2-A9RU	0	391	female	90	0	infiltrating ductal carcinoma	0	Her2	Tumor	6.410276343	7.271942265
TCGA-E9-A1N6	0	410	female	52	0	infiltrating ductal carcinoma	0	Normal	Normal	6.352699039	9.603394883
TCGA-E9-A1N9	0	399	female	58	0	nixed histology (please specify)	0	Normal	Normal	7.340971334	9.27686519
TCGA-E9-A1NC	0	708	female	61	0	nixed histology (please specify)	0	Basal	Tumor	6.359184844	8.026451021
TCGA-E9-A1NE	0	420	female	28	0	infiltrating ductal carcinoma	0	Basal	Tumor	6.307685671	8.308863384
TCGA-E9-A1NG	0	656	female	62	0	infiltrating ductal carcinoma	0	LumA	Tumor	6.322138795	8.780798201
TCGA-E9-A1R6	0	339	female	63	0	infiltrating ductal carcinoma	0	LumB	Tumor	6.617786172	7.282402688
TCGA-E9-A1R7	0	645	female	64	0	infiltrating ductal carcinoma	0	LumB	Tumor	6.407433059	7.422126644
TCGA-E9-A1RA	0	608	female	48	0	infiltrating ductal carcinoma	0	LumA	Tumor	6.355355212	7.636765466
TCGA-E9-A1RB	0	633	female	40	0	infiltrating ductal carcinoma	0	Normal	Normal	6.322726151	10.47662535
TCGA-E9-A1RC	0	1224	female	56	0	nixed histology (please specify)	0	LumB	Tumor	6.405958683	7.735346691
TCGA-E9-A1RG	0	647	female	62	0	infiltrating ductal carcinoma	0	LumB	Tumor	6.361980565	7.132389989
TCGA-E9-A1RI	0	425	female	43	0	nixed histology (please specify)	0	Normal	Normal	6.346816421	10.33797819
TCGA-E9-A22E	0	392	female	56	0	infiltrating ductal carcinoma	0	LumB	Tumor	6.337425028	8.536274847
TCGA-E9-A248	0	59	female	51	0	infiltrating ductal carcinoma	0	Basal	Tumor	6.707977955	7.309269648
TCGA-E9-A2JS	0	540	female	72	0	infiltrating lobular carcinoma	0	LumB	Tumor	6.675932136	6.86430256
TCGA-E9-A3X8	0	478	female	48	0	infiltrating lobular carcinoma	0	Normal	Tumor	6.518749595	8.359829854
TCGA-E9-A5FL	0	24	female	65	0	metaplastic carcinoma	0	Basal	Tumor	6.561947227	8.735221631
TCGA-E9-A6HE	0	468	female	45	0	infiltrating lobular carcinoma	0	LumB	Tumor	6.34668274	7.592127118
TCGA-EW-A1IY	0	258	female	38	0	infiltrating ductal carcinoma	0	LumB	Tumor	6.50821278	8.231160591
TCGA-EW-A1J1	0	575	female	38	0	infiltrating ductal carcinoma	0	LumA	Tumor	6.317664391	6.738949805
TCGA-EW-A1OY	0	908	female	63	0	infiltrating ductal carcinoma	0	LumB	Tumor	6.368545979	7.087107146
TCGA-EW-A1P4	0	907	female	43	0	medullary carcinoma	0	Basal	Tumor	6.628378447	8.871043963
TCGA-EW-A1P6	0	562	female	64	0	infiltrating ductal carcinoma	0	LumB	Tumor	6.373645777	7.947013351
TCGA-EW-A1P7	0	915	female	59	0	infiltrating ductal carcinoma	0	Basal	Tumor	6.813171366	8.342146798

TCGA-EW-A1PC	0	187	female	66	0	infiltrating ductal carcinoma	0	Her2	Tumor	6.441255377	6.963846729
TCGA-EW-A1PE	0	320	female	56	0	infiltrating ductal carcinoma	0	LumB	Tumor	6.322574845	7.13049636
TCGA-EW-A1PF	0	439	female	50	0	infiltrating ductal carcinoma	0	LumA	Tumor	6.330437499	7.908837896
TCGA-EW-A2FR	0	1309	female	59	0	infiltrating ductal carcinoma	0	Her2	Tumor	6.50529872	8.647017013
TCGA-EW-A2FV	0	428	female	39	0	other specify	0	Basal	Tumor	6.31658893	8.060705466
TCGA-EW-A2FW	0	412	female	52	0	other specify	0	LumB	Tumor	6.340280483	7.386726377
TCGA-EW-A3U0	0	532	female	61	0	infiltrating ductal carcinoma	0	Basal	Tumor	6.339563036	7.453501459
TCGA-EW-A6S9	0	463	female	34	0	infiltrating ductal carcinoma	0	Her2	Tumor	6.407566919	7.527273284
TCGA-GI-A2C9	0	3342	female	58	0	infiltrating ductal carcinoma	0	Basal	Tumor	6.966423688	9.061590477
TCGA-GI-A2C9	0	3342	female	58	0	infiltrating ductal carcinoma	0	Normal	Normal	6.323402742	9.291144943
TCGA-GM-A2DF	0	2155	female	53	0	infiltrating ductal carcinoma	0	Basal	Tumor	6.774516207	7.422274254
TCGA-GM-A2DH	0	2193	female	58	0	infiltrating ductal carcinoma	0	Her2	Tumor	7.413044356	7.117768936
TCGA-GM-A3NW	0	3361	female	63	0	infiltrating lobular carcinoma	0	Normal	Tumor	6.338853803	7.467960555
TCGA-GM-A3XL	0	2108	female	49	0	infiltrating ductal carcinoma	0	Basal	Tumor	6.6237156	6.491433878
TCGA-LD-A66U	0	422	female	44	0	infiltrating lobular carcinoma	0	LumA	Tumor	6.336586887	7.771430242
TCGA-LD-A74U	0	402	female	79	0	other specify	0	Normal	Tumor	6.407061794	7.809224873
TCGA-LL-A442	0	889	female	56	0	infiltrating ductal carcinoma	0	LumA	Tumor	6.313776868	6.313776868
TCGA-LL-A5YO	0	440	female	50	0	infiltrating ductal carcinoma	0	Her2	Tumor	6.576143158	7.419521886
TCGA-LL-A6FR	0	489	female	50	0	infiltrating ductal carcinoma	0	Basal	Tumor	6.498695691	7.618518544
TCGA-LL-A73Y	0	477	female	67	0	infiltrating ductal carcinoma	0	Basal	Tumor	7.769647499	9.164086284
TCGA-LL-A740	0	441	female	61	0	infiltrating ductal carcinoma	0	Her2	Tumor	6.858212855	8.118472878
TCGA-LL-A8F5	0	596	female	61	0	infiltrating ductal carcinoma	0	Basal	Tumor	6.37071411	7.07617051
TCGA-LL-A9Q3	0	532	female	69	0	infiltrating lobular carcinoma	0	LumA	Tumor	6.381370692	7.282276715
TCGA-LQ-A4E4	0	849	female	73	0	infiltrating lobular carcinoma	0	LumB	Tumor	6.44156525	7.446960834
TCGA-OL-A5RU	0	1219	female	63	0	infiltrating ductal carcinoma	0	LumA	Tumor	6.349073024	7.735694484
TCGA-OL-A5RW	0	1106	female	40	0	infiltrating ductal carcinoma	0	Basal	Tumor	6.654108367	8.276882369
TCGA-OL-A5RX	0	878	female	51	0	infiltrating ductal carcinoma	0	Normal	Tumor	6.345303275	8.123980992
TCGA-OL-A5S0	0	620	female	66	0	infiltrating ductal carcinoma	0	Basal	Tumor	6.450292758	7.740981163
TCGA-OL-A66H	0	812	female	74	0	mucinous carcinoma	0	LumA	Tumor	6.348524557	7.506560694
TCGA-OL-A66I	0	714	female	36	0	infiltrating ductal carcinoma	0	Basal	Tumor	6.56229	8.483312907
TCGA-OL-A66J	0	1996	female	80	0	infiltrating lobular carcinoma	0	LumA	Tumor	6.351737533	8.11773544
TCGA-OL-A66O	0	528	female	39	0	infiltrating ductal carcinoma	0	LumB	Tumor	6.345431636	7.242286381
TCGA-OL-A66P	0	428	female	75	0	infiltrating ductal carcinoma	0	Her2	Tumor	6.655375296	7.891221369
TCGA-OL-A6VO	0	858	female	43	0	infiltrating ductal carcinoma	0	Basal	Tumor	7.92429187	7.503256117
TCGA-OL-A6VR	0	1220	female	48	0	infiltrating ductal carcinoma	0	LumA	Tumor	6.323032181	7.638365273

TCGA-PL-A8LV	0	-7	female	54	0	medullary carcinoma	0	Basal	Tumor	7.143472889	10.38466822
TCGA-PL-A8LX	0	5	female	35	0	infiltrating ductal carcinoma	0	LumA	Tumor	6.437840552	7.676853203
TCGA-S3-A6ZF	0	502	female	64	0	infiltrating ductal carcinoma	0	LumB	Tumor	6.441834287	7.645617625
TCGA-S3-A6ZG	0	399	female	71	0	infiltrating lobular carcinoma	0	LumB	Tumor	6.321895016	7.211984488
TCGA-S3-A6ZH	0	515	female	29	0	infiltrating ductal carcinoma	0	Her2	Tumor	6.327928443	7.360962525
TCGA-S3-AA0Z	0	511	female	63	0	infiltrating ductal carcinoma	0	Basal	Tumor	6.396496648	7.035567556
TCGA-UL-AAZ6	0	518	female	73	0	infiltrating ductal carcinoma	0	Her2	Tumor	7.102024888	7.734808846
TCGA-V7-A7HQ	0	2033	female	75	0	infiltrating ductal carcinoma	0	LumA	Tumor	6.63288315	7.443415819
TCGA-WT-AB44	0	883	female	77	0	infiltrating lobular carcinoma	0	LumA	Tumor	6.429106929	7.915486374
TCGA-XX-A89A	0	488	female	68	0	infiltrating lobular carcinoma	0	Normal	Tumor	6.7286042	8.548356963
TCGA-Z7-A8R5	0	3287	female	61	0	infiltrating lobular carcinoma	0	Normal	Tumor	6.46963335	7.10323772
TCGA-Z7-A8R6	0	3256	female	46	0	infiltrating lobular carcinoma	0	LumB	Tumor	6.40784244	7.147810947
