# Supplementary figures and images for: FOXA2-Interacting FOXP2 Prevents Epithelial-Mesenchymal Transition of Breast Cancer Cells by Stimulating E-Cadherin and PHF2 Transcription
Source: Front Oncol. 2021 Feb 25;11:605025. doi: 10.3389/fonc.2021.605025 (PMC7947682; doi:10.3389/fonc.2021.605025)

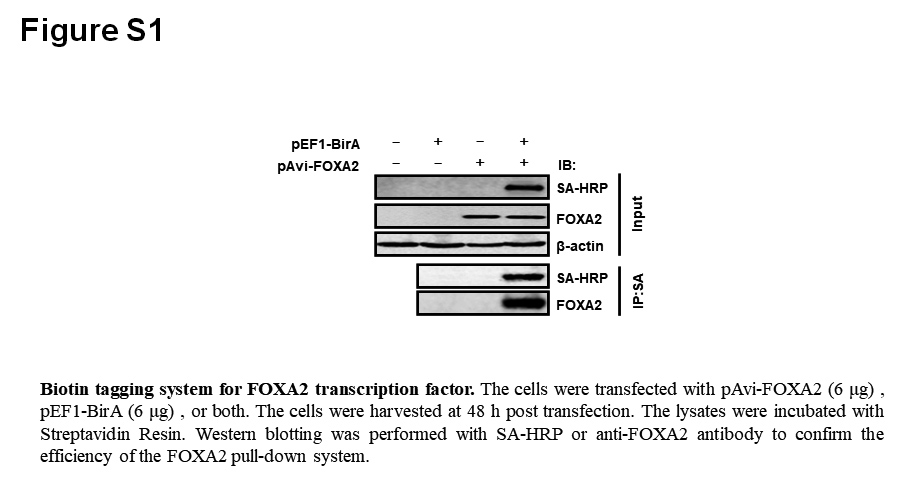

Supplement: Supplementary file 2 [file Image_1.jpeg]

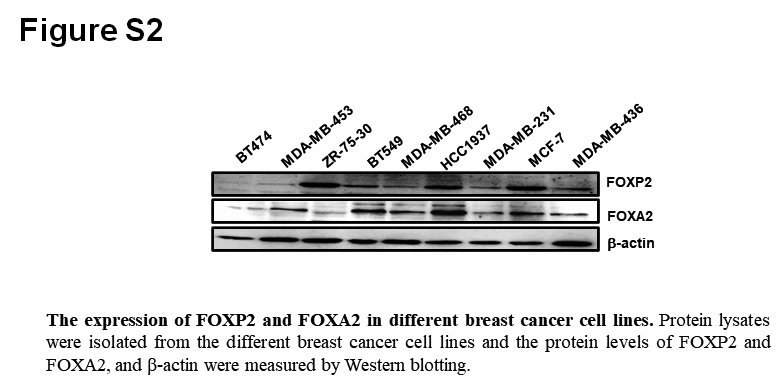

Supplement: Supplementary file 3 [file Image_2.jpeg]

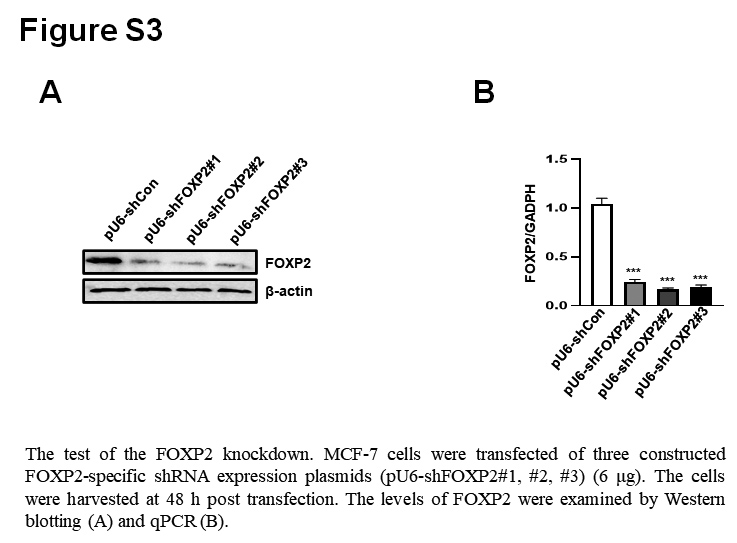

Supplement: Supplementary file 4 [file Image_3.jpeg]

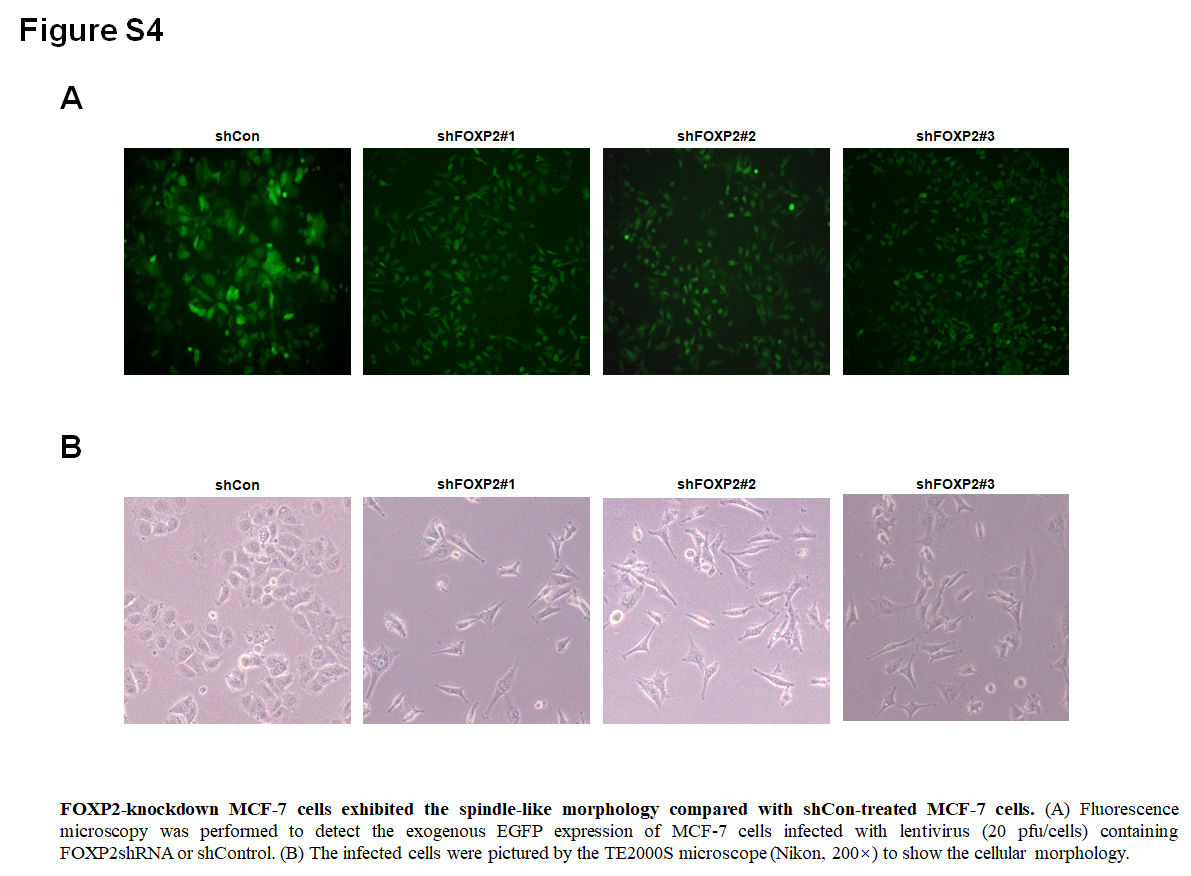

Supplement: Supplementary file 5 [file Image_4.jpeg]

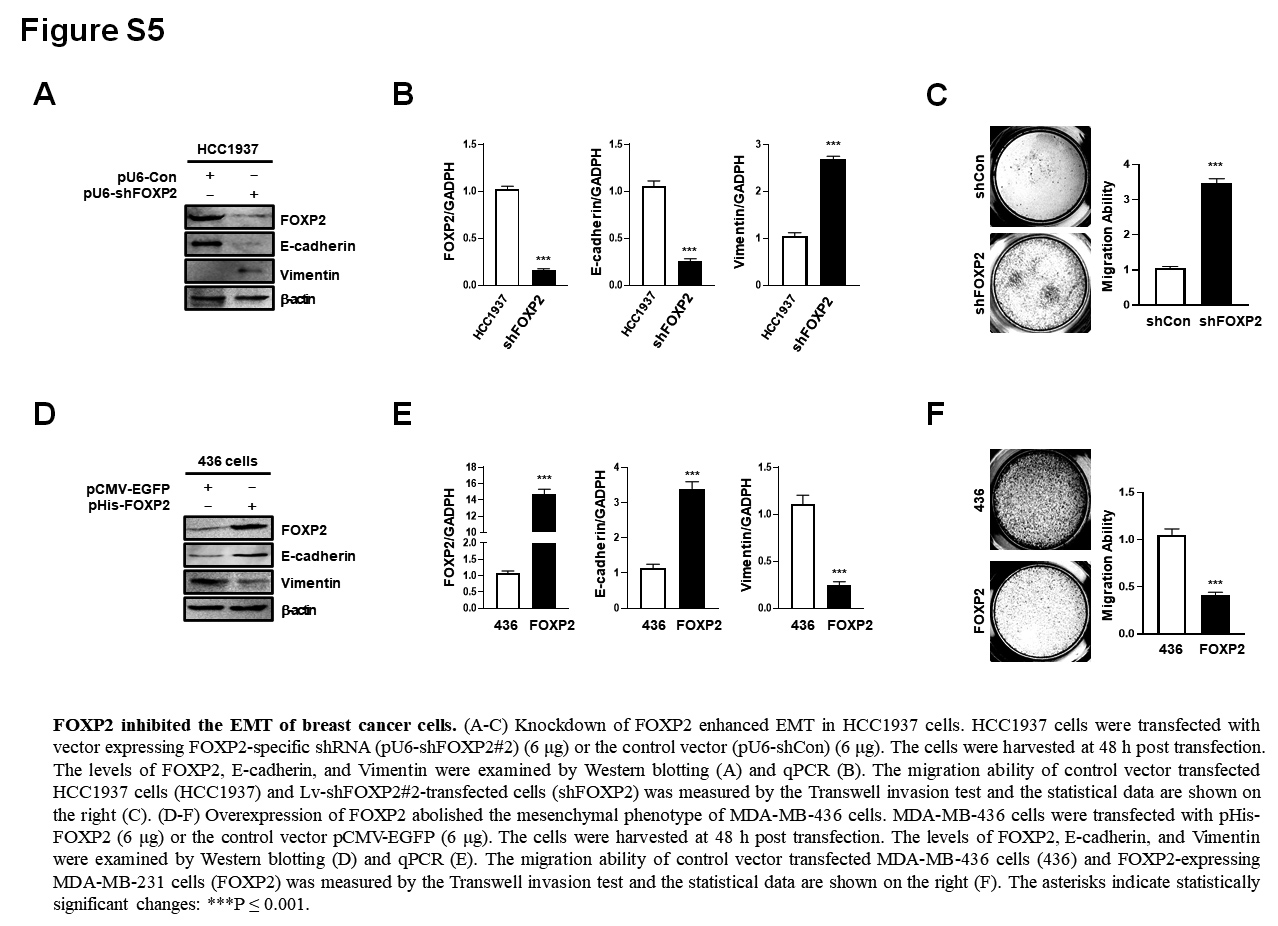

Supplement: Supplementary file 6 [file Image_5.jpeg]

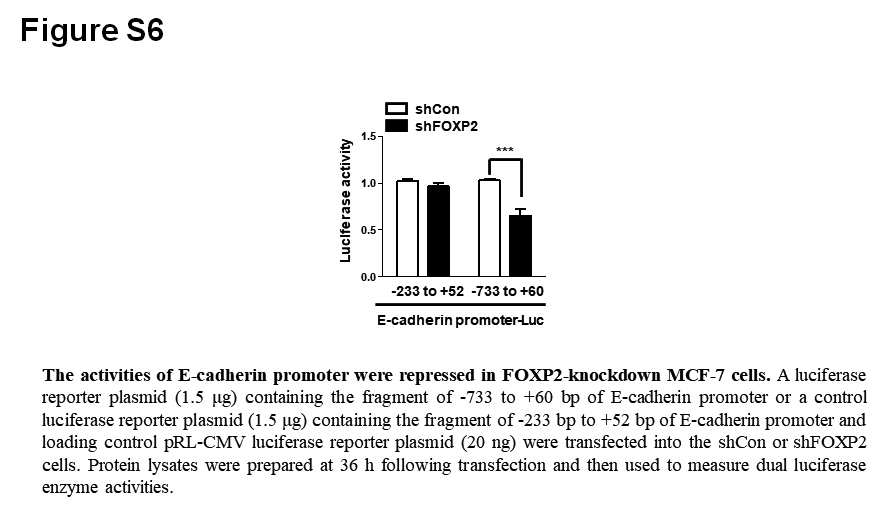

Supplement: Supplementary file 7 [file Image_6.jpeg]

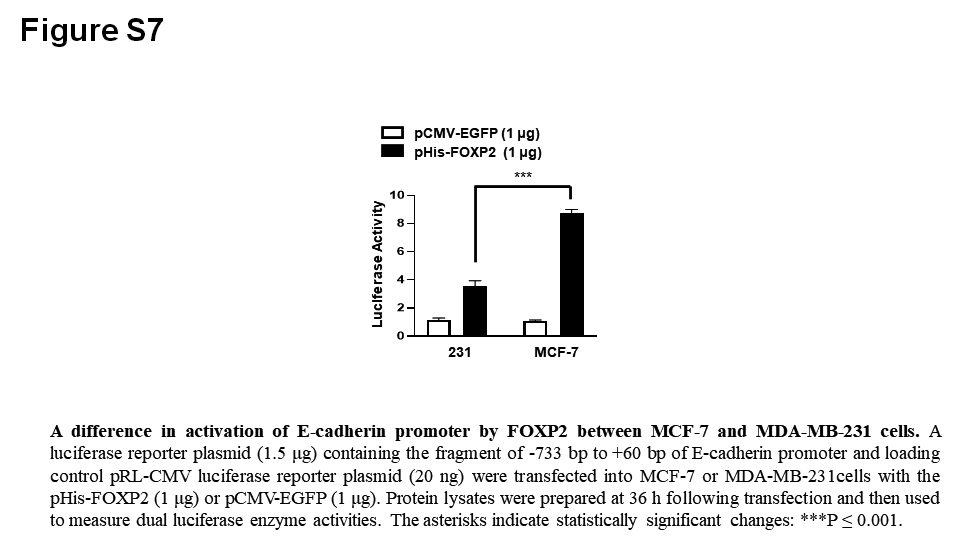

Supplement: Supplementary file 8 [file Image_7.jpeg]

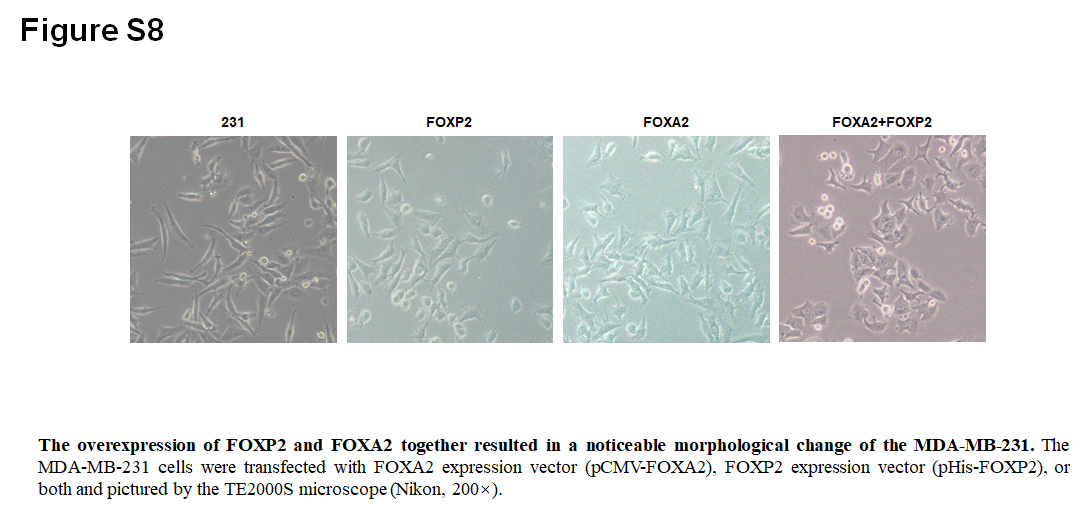

Supplement: Supplementary file 9 [file Image_8.jpeg]

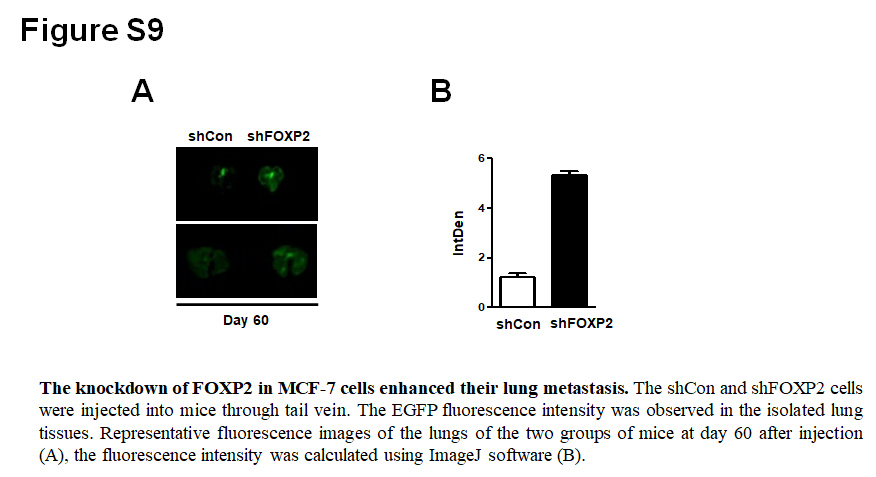

Supplement: Supplementary file 10 [file Image_9.jpeg]

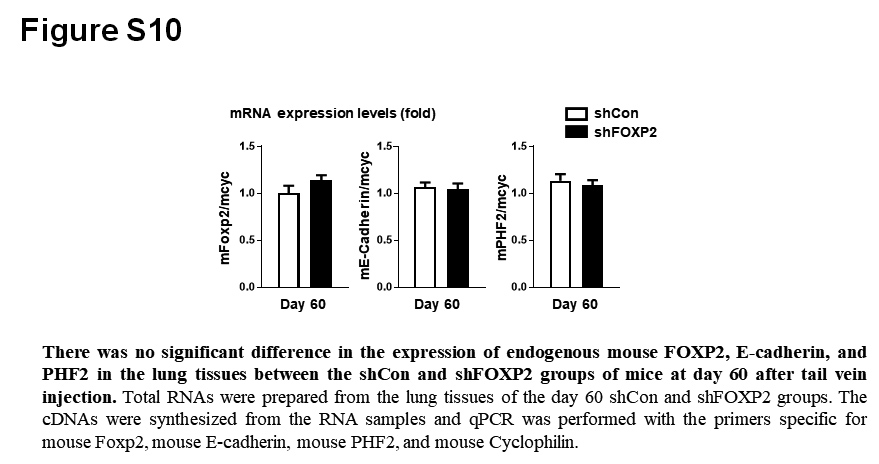

Supplement: Supplementary file 11 [file Image_10.jpeg]

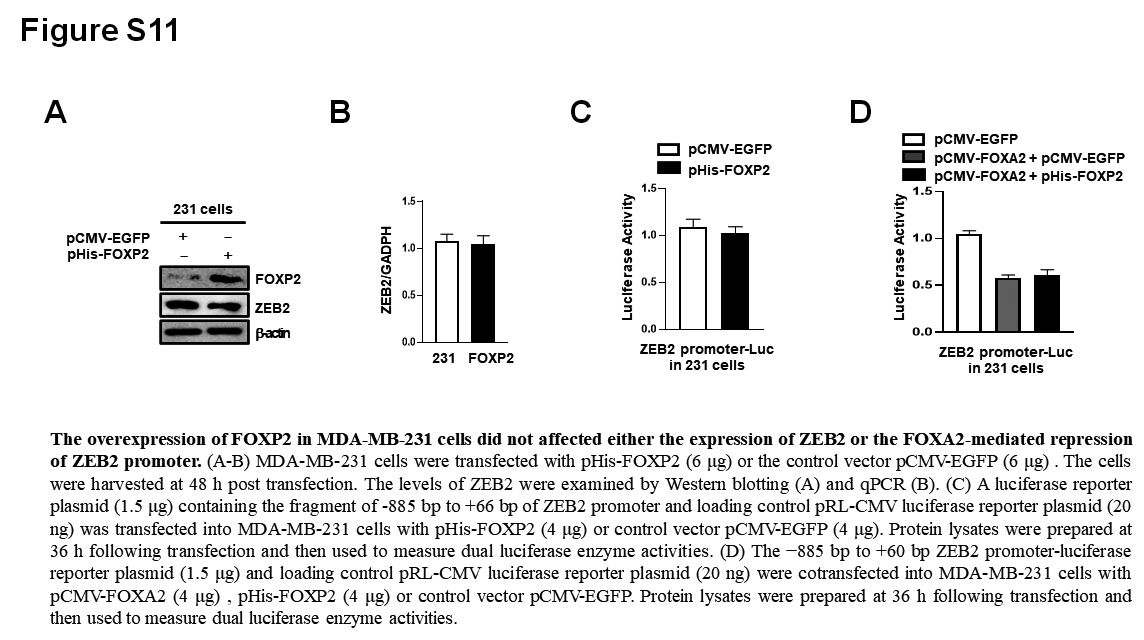

Supplement: Supplementary file 12 [file Image_11.jpeg]
